# Effectiveness of a blended in-person and online parenting programme in reducing violence against children in rural Thailand: a cluster randomised controlled trial

**DOI:** 10.1016/j.lansea.2026.100789

**Published:** 2026-05-28

**Authors:** Wilaiwan Pongpaew, Amalee McCoy, Sombat Tapanya, Jamie M. Lachman, Frances Gardner, G.J. Melendez-Torres, Qing Han, Piya Hanvoravongchai

**Affiliations:** a Department of Preventive and Social Medicine, Faculty of Medicine, Chulalongkorn University, Rama IV Road, Bangkok 10330, Thailand; b Department of Family Medicine, Faculty of Medicine, Chiang Mai University, Inthawarorot Road, Sri Phum, Chiang Mai 50200, Thailand; c Peace Culture Foundation, 155 Soi 6, Suan Luang Village, Baan Waen, Hang Dong, Chiang Mai 50230, Thailand; d Department of Social Policy and Intervention, University of Oxford, 32 Wellington Square, Oxford, OX1 2ER, UK; e Centre for Social Science Research, University of Cape Town, Cape Town, South Africa; f Parenting for Lifelong Health, Oxford, United Kingdom; g University of Exeter Medical School, St Luke’s Campus, South Cloisters, Heavitree Road, Exeter, EX1 2LU, UK; h Institute of Population Research, Peking University, Beijing, China; i Saw Swee Hock School of Public Health, National University of Singapore, 12 Science Drive 2, Singapore 117549, Singapore

**Keywords:** Violence against children, Child maltreatment, Positive parenting

## Abstract

**Background:**

Violence against children remains a substantial public health concern in Thailand. We evaluated whether a blended in-person and online messaging-based parenting programme could reduce child maltreatment.

**Methods:**

We conducted a parallel, two-arm, cluster-randomised, assessor-blinded trial in 12 sub-district health-promoting hospitals in Udon Thani, Thailand. Primary caregivers aged 18 years or older living with a child aged 2–17 were enrolled and randomised 1:1 to intervention or control (n = 240; approximately 20 per cluster). The nine-week intervention combined two brief in-person meetings with facilitated LINE™ (a widely used messaging application in Thailand) group chats. The primary outcome was the frequency of physical or emotional abuse in the past four weeks, assessed at one-month post-programme. Analyses were conducted on an intention-to-treat (ITT) basis using mixed-effects negative binomial models. Registered with the Open Science Framework (May 18, 2024; DOI 10.17605/OSF.IO/AB7GR; protocol available at https://osf.io/ysrku/overview?view_only=f0165cd340ef40e7ba2b0c5c79b6cbbf).

**Findings:**

Between 18 May and 2 June 2024, 240 caregivers were enrolled (120 per arm; 97.1% women). One-month follow-up was 90.0% (108/120) in the intervention and 91.7% (110/120) in the control arms. Overall child maltreatment showed no significant differences between groups (incidence rate ratio [IRR] 1.46, 95% confidence interval [CI] 0.83–2.57). No differences were observed for physical abuse (IRR 0.70, 95% CI 0.30–1.62). The relative estimates for emotional abuse was higher in the intervention arm (IRR 2.57, 95% CI 1.27–5.20).

**Interpretation:**

Results suggest no reductions in child maltreatment in the intervention group at one-month follow-up. The higher estimate for emotional abuse may reflect differential reporting or changes in awareness between groups. These findings suggest areas for refinement in intervention content and delivery. Future trials should target higher-risk participants and assess longer-term outcomes.

**Funding:**

The LEGO Foundation, Oak Foundation, and World Childhood Foundation via the Global Parenting Initiative.

**Research in context**

**Evidence before this study**

We reviewed global, regional and Thailand-specific evidence on parenting programmes, including those developed by Parenting for Lifelong Health (PLH) and similar programmes. Across randomised and quasi-experimental studies, such interventions generally yield small-to-moderate improvements in reducing harsh parenting and enhancing positive parenting. In Southeast Asia, however, robust trials remain limited. In Thailand, earlier evaluations of PLH for Young Children (PLH-YC) demonstrated improvements in parenting and child behaviour in public-service settings. Evidence on more cost-efficient, hybrid messaging-based programmes that blend in-person and online delivery within routine services is limited, motivating this cluster-randomised evaluation.

**Added value of this study**

This trial provides cluster-randomised evidence on the effectiveness of a hybrid in-person and messaging-based parenting programme delivered by public health workers within routine primary care in rural Thailand. This delivery model has not previously been evaluated in this setting. The null findings on overall child maltreatment, reported transparently alongside a significant reduction in the severity of challenging child behaviour, contribute to the growing literature on the conditions under which digital and hybrid parenting programmes can and cannot produce measurable change. Implementation data on attendance, content fidelity, and facilitator engagement offer actionable insights for future programme refinement. The predominantly grandparent caregiving sample further adds contextually distinct evidence rarely captured in the parenting intervention literature.

**Implications of all the available evidence**

Parenting interventions delivered by trained facilitators via in-person group sessions and online messaging show potential but may not be sufficient to reduce child maltreatment. This facilitator-led hybrid approach differs from digital self-guided or fully online programmes, as it relies on structured interaction and peer discussion rather than independent engagement. Effective scale-up will require not only stronger content and facilitator training, but also deliberate strategies to address digital access and literacy constraints in rural communities. Taken together with previous evidence, digital and hybrid parenting programmes should be considered complementary rather than stand-alone solutions, with future trials focusing on longer-term outcomes, implementation strategies, and precautions against unintended harms.

## Introduction

Violence against children remains a serious global public health challenge. Beyond its immediate harm, violence against children can disrupt early developmental processes, with evidence linking toxic stress exposure to long-term difficulties in neurodevelopment, emotional regulation, and social functioning.^1^^,^^2^ Parents and other primary caregivers are the most common perpetrators of violence against children, particularly in the form of harsh discipline within the home. Recent estimates suggest that more than one billion children aged 2–17 years experience some form of violence each year worldwide, with the largest absolute burden in populous regions such as Asia and Africa.^1^ Exposure to child maltreatment is associated with a wide range of adverse lifelong consequences, including poor mental health, impaired educational attainment, and increased risk of violence later in life, contributing to substantial social and economic costs.^2^ At the population level, these individual harms translate into substantial intergenerational and societal burdens, reinforcing cycles of disadvantage and inequity, particularly in low- and middle-income settings. These impacts underscore the need for prevention approaches that are both effective and scalable. In Thailand, over half of children (53.8% of those aged 1–14) experience violent discipline, underscoring the urgent need for population-level prevention approaches.^3^ The World Health Organization identifies structured parenting programmes as a core evidence-based strategy for violence prevention, with evidence showing they can reduce the prevalence of child maltreatment by 20%–50%.^4^ However, effects are often modest and may vary by population, delivery format, and baseline risk, particularly in universal community samples where levels of maltreatment may be relatively low at enrolment. Parenting for Lifelong Health (PLH) programmes have shown benefits when delivered in face-to-face formats.^5^ However, these models typically rely on repeated group sessions facilitated by trained personnel, which can be resource-intensive and difficult to implement at scale within routine public health systems. Traditional face-to-face PLH delivery can also involve substantial operational costs, and workload demands for public health workers, further limiting scalability.^6^ These limitations have led to growing interest in alternative delivery models that retain core intervention components while reducing resource demands and improving accessibility. Messaging-based platforms may offer a scalable alternative for parenting support, particularly in settings such as Thailand where smartphone use is widespread and LINE™ is already well established in daily communication. However, there is still limited evidence on whether hybrid models that combine in-person contact with remote, facilitator-supported messaging can produce measurable reductions in child maltreatment. Moreover, findings from digital parenting interventions have been inconsistent, raising concerns about participant engagement, the adequacy of intervention exposure, and the sustainability of behaviour change.^7^, ^8^, ^9^ Against this background, the ParentChat programme was developed as a hybrid model that combined two in-person meetings with seven facilitated sessions delivered through LINE™, a widely used messaging application in Thailand that supports text, image, audio, and group-based communication. The intervention was designed to balance scalability with fidelity by retaining facilitator support and peer interaction while reducing the intensity and logistical demands of traditional group-based delivery. Against this background, this trial aimed to evaluate whether a hybrid facilitator-led parenting programme, combining two in-person sessions with seven weeks of structured LINE™-based group support, could produce measurable reductions in caregiver-reported child maltreatment in a rural Thai primary health care setting.

## Methods

### Study design and participants

We conducted a parallel, two-arm, cluster-randomised, assessor-blinded trial in 12 sub-district Health Promoting Hospital (HPHs) in Udon Thani, Thailand. Udon Thani is a predominantly rural province in northeastern Thailand with demographic and socioeconomic characteristics broadly comparable to many rural provinces nationally. The study setting was selected because the HPH network provides the primary platform for community-based parenting and child health services, making it a relevant context for programme implementation within routine primary care. Clusters were defined at the sub-district level to reflect the rural primary health context and to minimise contamination between study arms. The trial adhered to the CONSORT extension for cluster randomised trials (CONSORT-CRTs). The checklist and full protocol are provided in the Supplementary Appendix.

After two-stage cluster sampling, 12 sub-districts were randomised 1:1 to intervention or control. Participants were primary caregivers (≥18 years) with a child aged 2–17. No screening threshold for violence exposure or child behavioural problems was applied during recruitment. This approach reflects a universal prevention strategy targeting caregivers before harmful practices become established. Baseline levels of physical and emotional abuse were measured using the ICAST instrument at enrolment for evaluation purposes rather than eligibility determination. Participant sex (female or male) was self-reported at baseline. Including caregivers not currently reporting maltreatment may reduce detectable effect sizes while increasing relevance for population-level implementation. Key eligibility criteria included: being a primary caregiver aged 18 years or older with a child aged 2–17; having regular contact with the child (living together for at least four nights per week); having access to an internet-enabled smartphone and being willing and available to join a LINE-based support group over the nine-week programme period. Exclusion criteria included severe mental health impairment or prior completion of a similar parenting programme. Twenty eligible caregivers were recruited per cluster (planned n = 240) via HPH records and staff referrals.

Clusters were randomised 1:1 to intervention or control after baseline assessment completion. An independent statistician generated the allocation list using a computer-generated random number sequence with no stratification or blocking. The allocation sequence was concealed in a password-protected file accessible only to the statistician. After baseline data was locked, group assignments were released to the research manager.

Blinding of facilitators and participants was not possible due to the behavioural nature of the intervention. Bias was reduced through allocation concealment and assessor blinding. Field data collectors and trial statisticians were blinded to group assignment (datasets were labelled “1” or “0”). Caregivers were told the programme would be offered at different times to different sub-districts, limiting expectancy effects.

### Procedures

Six local nurse practitioners attended a four-day course and received weekly Zoom coaching for fidelity. The nine-week intervention (ParentChat) combined two face-to-face meetings (in Weeks 1 and 9) with seven weekly remote sessions. Remote sessions were delivered through facilitated LINE™ group chats, covering seven core modules including positive discipline and safety. Facilitators provided support and promoted home practice (details in Supplementary Table S1). Control clusters used identical LINE™ groups with no training content. To minimise contamination, the intervention was delivered at the cluster level, with separate LINE™ groups and facilitators assigned to each cluster. Participants were encouraged not to share intervention materials across groups, and group activity was monitored by the research team. Both groups accessed usual maternal and child health services (e.g., immunisation, developmental monitoring, and general outpatient care). No structured parenting programmes comparable to the intervention were routinely available in the study area during the trial period.

Data were collected at baseline (Week 0) and one-month post-programme (Week 14) via blinded field visits. Brief telephone assessments captured primary outcome trends in Weeks 4 and 8. Serious adverse event monitoring was built into weekly facilitator check-ins (reporting to A.M. and S.T.). Implementation quality was tracked using facilitator checklists (target ≥ 80% content coverage).

### Outcomes

Outcomes were assessed at baseline, during programme delivery (Weeks 4 and 8), and one-month post-intervention. The Week 4 and Week 8 assessments were included to capture intermediate behavioural changes during programme delivery, while the one-month post-intervention assessment represented the prespecified primary endpoint to evaluate short-term programme effects. All instruments, scoring rules, and assessment time points were pre-specified in the protocol. All instruments were forward-translated into Thai, back-translated and cognitively tested for cultural relevance.

The single primary outcome was the caregiver-reported frequency of overall child maltreatment over the past four weeks, measured with the four-item ISPCAN Child Abuse Screening Tool-Trial Parent version (ICAST-TP^10^; α = 0.69). Items are rated on an eight-point scale (0 = “Never” to 8 = “8 or more”), totalling 0–32. We also assessed the component subscales (physical abuse, two items; and emotional abuse, two items) as proximal outcomes. Higher scores indicate frequent maltreatment.

Proximal outcomes reflecting immediate parenting changes were assessed at baseline and one-month post-intervention: (1) Positive parenting and involvement (measured with the adapted Alabama Parenting Questionnaire [APQ]^11^; five items; α = 0.88); and (2) Parental stress (measured by the Parental Stress Scale [PSS]^12^; five items; α = 0.65). For brief telephone assessments in Weeks 4 and 8, two APQ items (fun time/praise) and one PSS item (child as source of stress) were used. Higher APQ scores indicate more positive interactions, while higher PSS scores reflect greater stress.

Secondary outcomes were classified as all pre-specified non-primary or proximal endpoints. At baseline and one-month post-intervention, we assessed: attitudes towards corporal punishment (MICS^13^); child behavioural problems (CABI^14^; α = 0.89); the single “most challenging child behaviour” (Three Problem Scale-Parent Report^15^); parental mental health (DASS-21^16^; α = 0.85); caregiver exposure to intimate partner violence (IPV) and intimate partner coercion (WHO Multi-Country Questionnaire on Women’s Health and Domestic Violence against Women^17^; α = 0.68); child/adolescent exposure to risks (adapted from ParentApp for Teens in Tanzania and ParentText in South Africa; α = 0.97); online safety (Global Kids Online^18^; α = 0.96); Child learning and development (MICS^13^; α = 0.92); and parental support of education (adapted from Ceballos' Family^19^; α = 0.71).

### Statistical analysis

All analyses followed a prespecified plan finalised before database lock and adhered to CONSORT guidelines for cluster-randomised trials.^20^ Analyses were conducted on an intention-to-treat (ITT) basis, including all randomised caregivers with at least one post-baseline measurement. We used Stata/SE 19.5 (StataCorp LLC, 2025) for all computations. Longitudinal outcomes were analysed using mixed-effects regression models to account for clustering and repeated measurements. Models included random intercepts for cluster and participant, and fixed effects for group, time (categorical), and their interaction (yielding the difference-in-differences treatment estimate). For count outcomes (e.g., ICAST-TP and APQ) that were overdispersed and zero-inflated, negative binomial mixed models were used, with results reported as incidence rate ratios (IRRs) and 95% confidence intervals. Approximately normal outcomes (e.g., PSS, DASS-21) were analysed using linear mixed-effects models, with results reported as mean differences (β) with 95% confidence intervals. All tests were two-sided with a significance level of 0.05. P-values for secondary and exploratory outcomes were not adjusted. Sensitivity analyses were conducted to assess the robustness of findings. For outcomes with excess zeros, prespecified mixed-effects logistic regression models (any vs. none) were estimated. Exploratory analyses using population-averaged generalised estimating equation (GEE) models examined results under an alternative modelling framework (Supplementary Table S3).

Sample size was calculated for a cluster-randomised design using a conservative design effect of 2.0, based on published ranges of 1–5.5.^21^ This required a sample of 240 caregivers (120 per arm, 20 per cluster across 12 clusters) to account for clustering effects, doubling a simple random sample of 120. Post-hoc analysis revealed a low Intraclass Correlation Coefficient (ICC) of 0.00 and an actual Design Effect of 1.0, confirming that the trial was adequately powered despite the limited number of 12 clusters. Missing data were handled using full-information maximum likelihood under a missing-at-random assumption.

### Patient and public involvement (PPI) statement

Caregivers were not involved in the design or reporting of this research. The study involved key stakeholders and public health officials in the programme adaptation and delivery, providing logistical support and facilitating integration into the routine public health services.

### Ethics approval

Ethical approval was obtained from the Institutional Review Board, Faculty of Medicine, Chulalongkorn University (COA 0583/2024; IRB 0818/66). Written informed consent was obtained from all participants. For participants with limited literacy, consent information was read aloud and oral consent was documented by the research team, as specified in the study protocol.

### Role of the funding source

This study was part of the Global Parenting Initiative, funded by the LEGO Foundation, Oak Foundation, and World Childhood Foundation. The funders had no role in the study design, data collection, data analysis, data interpretation, report writing, or the decision to submit for publication. The corresponding author retained full access to all data and had final responsibility for the submission decision.

## Results

### Participant flow and baseline characteristics

**Fig. 1:**
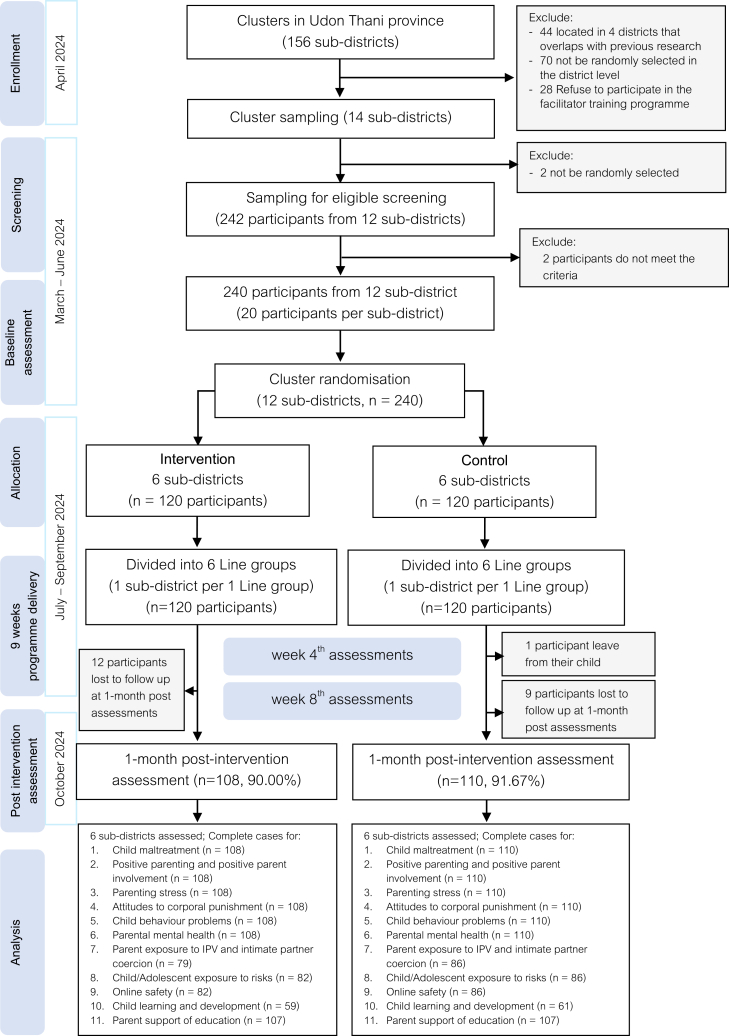
Trial profile.

Between March and April 2024, 12 sub-districts were selected, and 240 caregivers were cluster-randomised 1:1 to ParentChat intervention (n = 120) or control (n = 120). Of the 240 enrolled caregivers, 218 (90.8%) completed the one-month post-intervention assessment. Twenty-two participants were lost to follow-up (12 intervention; 10 control), resulting in missing outcome data at the primary endpoint. Participant flow is detailed in Fig. 1. Baseline caregivers were generally well balanced between groups (Table 1), except for the proportion of grandparents as primary caregivers. Sensitivity analyses confirmed this imbalance did not affect primary outcome conclusions.

**Table 1:** Socio-demographic characteristics of caregivers and children at baseline.

Variables	Control	Intervention	Overall sample
	n = 120	n = 120	n = 240
Caregiver variables			
Age, year, mean (SD) [Range]	47.31 (10.64) [24–69]	50.43 (10.67) [26–72]	48.87 (10.75) [24–72]
Female, n (%)	116 (96.67)	117 (97.50)	233 (97.08)
Mainly speaks Isan language at home, n (%)	98 (81.67)	102 (85.00)	200 (83.33)
Can read easily, n (%)	107 (89.17)	105 (87.50)	212 (88.33)
Ethnicity–Thai Siamese, n (%)	96 (80.00)	87 (72.50)	183 (76.25)
Thai citizenship, n (%)	120 (100.00)	120 (100.00)	240 (100.00)
Education level			
Primary school, n (%)	19 (15.83)	36 (30.00)	55 (22.92)
Secondary school or Vocational school, n (%)	94 (78.33)	79 (65.83)	168 (70.00)
University level, n (%)	7 (5.83)	5 (4.17)	12 (5.00)
Employment			
Not working, n (%)	11 (9.17)	22 (18.33)	33 (13.75)
Farmer, n (%)	56 (46.67)	58 (48.33)	114 (47.50)
Formal sector—Government, n (%)	1 (0.83)	3 (2.50)	4 (1. 67)
Private employee, n (%)	2 (1.67)	1 (0.83)	3 (1.25)
Private enterprise/own account, n (%)	12 (10.00)	7 (5.83)	19 (7.92)
General employee/unskilled labour, n (%)	25 (20.83)	14 (11.67)	39 (16.25)
Other	13 (10.83)	15 (12.50)	28 (11.67)
Child variables			
Age, mean (SD)	9.56 (4.23)	9.11 (4.42)	9.33 (4.32)
Girl, n (%)	53 (44.17)	52 (43.33)	105 (43.75)
School enrollment^a^, n (%)	116 (96.67)	120 (100.00)	236 (98.33)
Child relationship variables			
Relationship to target child			
Grandmother/grandfather, n (%)	57 (47.50)	75 (62.50)	132 (55.00)
Biological mother, n (%)	56 (46.67)	39 (32.50)	95 (39.58)
Other relative, n (%)	7 (5.83)	6 (5.00)	13 (5.42)
Caregivers have other children in their care, n (%)	67 (55.83)	63 (52.50)	130 (54.17)
Number of other children in their care, mean (SD)	1.39 (0.76)	1.21 (0.48)	1.30 (0.64)
Household variables			
Number of adults in the household, mean (SD)	2.88 (1.19)	3.08 (1.27)	2.98 (1.23)
Absent of biological father at home, n (%)	74 (61.67)	85 (70.83)	159 (66.25)
Absent of biological mother at home, n (%)	54 (45.00)	59 (49.17)	113 (47.08)
Neither biological parent living in the household, n (%)	47 (39.17)	53 (44.17)	100 (41.67)
Total monthly household income			
≤5000 Baht (141 USD), n (%)	17 (14.17)	24 (20.00)	41 (17.08)
5001–15,000 Baht (141–423 USD), n (%)	75 (62.50)	64 (53.33)	139 (57.92)
15,001–30,000 Baht (423–846 USD), n (%)	17 (14.17)	25 (20.83)	42 (17.50)
More than 30,000 Baht (846 USD), n (%)	11 (9.17)	7 (5.83)	18 (7.50)
Children have other caregivers in the household, n (%)	101 (84.17)	108 (90.00)	209 (87.08)
Number of other caregivers, mean (SD)	1.86 (0.94)	2.04 (1.08)	1.95 (1.02)
Relationship to the head of household, n (%)			
Head	29 (24.17)	37 (30.83)	66 (27.50)
Wife/husband	58 (48.33)	50 (41.67)	108 (45.00)
Son/daughter	25 (20.83)	18 (15.00)	43 (17.92)
Other	8 (6.67)	15 (12.50)	23 (9.58)
Caregiver and child disability (physical functioning/cognitive or learning functioning)			
Adult disability, n (%)	4 (3.33)	6 (5.00)	10 (4.17)
Child disability, n (%)	0 (0.00)	3 (2.50)	3 (1.25)

SD = standard deviation; USD = United States dollars.

Data are presented as n (%) for categorical variables and mean (SD) for continuous variables.

a School also includes preschool, daycare, childcare, nursery school, and kindergarten.

The mean caregiver age was 48.87 years (SD 10.75); 97.08% (233/240) were women, and 55% (132/240) were grandparents. Children’s mean age was 9.33 years (SD 4.32). Follow-up at one-month post-programme was 90.00% (108/120) in the intervention arm and 91.67% (110/120) in the control arm. Interim assessments via telephone showed an assessment completion rate of 100% (120/120) in the intervention arm and 99.17% (119/120) in the control arm in Week 4, and 100% (120/120) and 99.17% (119/120), respectively, in Week 8. Thus, the primary-endpoint dataset comprised 218 complete cases, but all mixed-effects models analysed the full 240 participants with available repeated measures.

### Intervention delivery and fidelity

Fidelity indicators suggested that the programme was delivered largely as intended. Overall attendance across the nine-week programme averaged 77.31%, with high attendance at the in-person sessions (91.67%) and substantial engagement in LINE group chats (73.21%). Implementation quality was monitored using facilitator checklists. Mean reported content coverage was 95.18%, exceeding the predefined target of ≥80%.

### Primary outcome

Full results for all primary, proximal, and secondary outcomes are shown in Table 2. Child maltreatment outcomes were highly skewed, with median counts of zero incidents across nearly all timepoints. When combined into the overall maltreatment measure, no statistically significant group differences were observed (IRR 1.46, 95% CI 0.83–2.57).

**Table 2:** Primary, proximal, and secondary outcomes at one-month post-intervention.

Outcome	Group	Baseline	Post-test	Effect estimates	95% CI	Model type
		(Median, IQR or Mean, SD)				
		(n = 240)	(n_complete_)			
Child maltreatment (ICAST-TP)	Control	1 (0, 2)	0 (0, 1)	IRR 1.46	0.83–2.57	Negative binomial
	Intervention	1 (0, 2.5)	0 (0, 2)			
Physical abuse (subscale)	Control	0 (0, 0)	0 (0, 0)	IRR 0.70	0.30–1.62	Negative binomial
	Intervention	0 (0, 0.5)	0 (0, 0)			
Emotional abuse (subscale)	Control	0 (0, 2)	0 (0, 0)	IRR 2.57^a^	1.27–5.20	Negative binomial
	Intervention	1 (0, 2)	0 (0, 1.5)			
Positive parenting and involvement (APQ)	Control	33 (26, 38.5)	35 (27, 39)	IRR 0.997	0.93–1.07	Negative binomial
	Intervention	31.50 (24.5, 37)	33.00 (26, 37)			
Parenting stress (PSS)	Control	3.40 (2.66)	3.50 (2.47)	β −0.09	−1.10 to 0.92	Linear mixed
	Intervention	3.39 (2.67)	3.41 (2.50)			
Attitudes to corporal punishment (MICS: child discipline module)	Control	1 (0, 1)	1 (0, 1)	IRR 1.002	0.68–1.47	Poisson
	Intervention	1 (0, 1)	1 (0, 1)			
Child behaviour problems (CABI)	Control	3.03 (3.14)	2.68 (2.83)	β −0.87	−1.75 to 0.01	Linear mixed
	Intervention	4.83 (4.63)	3.49 (3.42)			
Intensity of the most challenging behaviour (Three Problem Scale-Parent Report)	Control	1.26 (1.62)	1.45 (1.65)	β −0.51^a^	−0.99 to −0.03	Linear mixed
	Intervention	1.91 (1.90)	1.59 (1.65)			
Parental mental health (DASS-21: depression subscale)	Control	1.12 (1.94)	0.95 (1.80)	β −0.001	−0.65 to 0.65	Linear mixed
	Intervention	1.42 (1.98)	1.23 (1.90)			
Parent exposure to IPV and intimate partner coercion (VAWI) (n = 181)^b^	Control	0 (0, 2)	0 (0, 3)	IRR 1.13	0.54–2.36	Negative binomial
	Intervention	1 (0, 5)	2 (0, 4)			
Child/adolescent exposure to risks (n = 185)^b^	Control	0 (0, 0)	0 (0, 0)	IRR 0.35	0.03–3.88	Negative binomial
	Intervention	0 (0, 0)	0 (0, 1)			
Online safety (n = 185)^b^	Control	0 (0, 0)	0 (0, 0)	IRR 1.24	0.20–7.46	Negative binomial
	Intervention	0 (0, 0)	0 (0, 0)			
Child learning and development (n = 131)^b^	Control	31 (20, 36)	29 (24, 36)	IRR 1.03	0.91–1.18	Negative binomial
	Intervention	26 (19, 33)	28 (22, 36)			
Parent support of education (n = 236)^b^	Control	14.60 (3.10)	15.02 (2.65)	β 0.20	−0.85 to 1.24	Linear mixed
	Intervention	13.71 (3.52)	14.38 (3.44)			

IRR = incidence rate ratio; CI = confidence interval; IQR = interquartile range; SD = standard deviation; β = regression coefficient from linear mixed-effects models; IPV = intimate partner violence; APQ = Alabama Parenting Questionnaire; CABI = Child and Adolescent Behavior Inventory; PSS = Parental Stress Scale.

a P < 0.05 (two-sided). Data are reported using Median (IQR) for count outcomes analysed using negative binomial or Poisson models and mean (SD) for approximately normal distributed outcomes analysed using linear mixed-effects models. The number of participants for each outcome is derived from the full sample (n = 240) and detailed in Fig. 1.

b The number of participants included in each analysis varies by outcome based on specific eligibility: IPV/Partner Coercion was assessed among caregivers with a partner (n = 181); child/adolescent exposure to risks and online safety were assessed among children aged ≥ 6 years (n = 185); child learning and development was assessed among children aged 2–10 years (n = 131); and parent support of education was assessed among children currently enrolled in school (n = 236).

### Proximal and secondary outcomes

For physical abuse, the median number of incidents was zero across all assessments in both groups. The proportion of caregivers reporting at least one incident remained low and stable over time. The mixed-effects model indicated no significant group differences (IRR 0.70, 95% CI 0.30–1.62). Prespecified sensitivity analyses using mixed-effects logistic regression (any vs. none) yielded similar conclusions (Supplementary Table S3). Exploratory population-averaged GEE models indicated a statistically significant reduction in physical abuse (IRR 0.46, 95% CI 0.24–0.90), although these analyses were not prespecified and should be interpreted cautiously (Supplementary Table S3).

For emotional abuse, median monthly incidents were zero in all periods except baseline, when the intervention group reported slightly higher levels. The proportion reporting ≥ 1 event fell in both groups but showed a greater, sustained decline in the control group at post-test (16.4% vs. 37.0%; Supplementary Fig. S1a). This difference resulted in a higher relative estimate for the intervention arm (IRR 2.57, 95% CI 1.27–5.20) and may reflect differential declines between groups rather than an absolute increase in emotional abuse.

Most proximal and secondary outcomes showed no significant group differences: positive parenting (IRR 0.997, 95% CI 0.93–1.07), parenting stress (β −0.09, 95% CI –1.10 to 0.92), attitudes towards corporal punishment (IRR 1.00, 95% CI 0.68–1.47), parental mental health (β −0.00, 95% CI –0.65 to 0.65), child learning and development (IRR 1.03, 95% CI 0.91–1.18), and parental support of education (β 0.20, 95% CI –0.85 to 1.24) all showed no detectable differences. However, the intensity of the most challenging child behaviour decreased modestly in the intervention group compared with control (β −0.51, 95% CI –0.99 to −0.03). Child behaviour problems showed a non-significant improvement (β −0.87, 95% CI –1.75 to 0.01). IPV and intimate partner coercion (IRR 1.22, 95% CI 0.54–2.36), child/adolescent exposure to risks (IRR 1.41, 95% CI 0.31–6.42), and online-safety (IRR 1.24, 95% CI 0.20–7.46) showed imprecise estimates.

### Other analyses and safety

Ancillary analyses also supported the robustness of the null findings. A mixed-effects ordinal logistic regression for attitudes toward corporal punishment yielded similar findings (OR 0.93, 95% CI 0.45–1.92), and adjustment for the baseline imbalance in grandparent caregiver status had minimal impact on the primary outcome estimates.

The exploratory analysis of ‘Showing care and respect’ found no significant impact on positive intimate partner interaction (IRR 1.31, 95% CI 0.87–1.97), detailed in Supplementary Table S2. Crucially, no adverse event was reported in either group throughout the trial period.

## Discussion

This study evaluated a blended digital parenting programme combining group sessions with remote online messaging support. These findings are relevant in the context of increasing use of digital platforms to support parenting interventions. Facilitators provided basic guidance during home visits prior to the first in-person session to help caregivers join and navigate the LINE™ groups, and additional support was offered when participants experienced technical difficulties. However, variation in digital literacy, particularly among older caregivers and grandparents, may still have influenced engagement with the messaging components and reduced the amount of intervention exposure received by participants.

This trial found no overall reduction in caregiver-reported child maltreatment. These findings are broadly consistent with several recent evaluations of brief parenting or digital parenting interventions in low-resource settings, which have reported mixed or modest effects on harsh discipline outcomes. Trials of Parenting for Lifelong Health programmes in Africa^22^ and Southeast Asia^5^^,^^23^ have demonstrated reductions in violent discipline in some contexts, although effects have varied depending on programme intensity, delivery format, and follow-up duration.^7^^,^^9^ Although both physical and emotional abuse declined in absolute terms, reductions were greater in the control arm, yielding a higher relative estimate for emotional abuse. These results indicate that the current programme version may not have been sufficiently intensive to reduce maltreatment or strengthen positive parenting behaviours.

Interpretation of the child maltreatment outcomes requires caution. Distributions of both physical and emotional abuse were highly skewed, with medians of zero incidents across most assessments, suggesting low baseline rates that limited room for measurable improvement. Exploratory analyses using population-averaged GEE models suggested a possible reduction in physical abuse. However, because these analyses were not prespecified and the primary mixed-effects models showed no significant effect, these results should be interpreted cautiously.

Baseline levels of reported violence were relatively low and somewhat lower than expected based on national prevalence estimates.^3^ This likely reflects the universal recruitment strategy used in the trial, which enrolled caregivers from community health services rather than targeting families already reporting maltreatment, thereby limiting the scope for measurable reductions during the short follow-up period.

Although emotional abuse appeared less reduced in the intervention group than the control, this pattern should not be interpreted as a true negative effect. Possible explanations include behavioural substitution,^24^ such as a shifting from physical punishment to verbal expressions of anger during a period of temporary emotional adjustment, and measurement reactivity, whereby increased self-awareness among caregivers exposed to the programme may have encouraged more complete disclosure of behaviours that might previously have been under-reported.^25^, ^26^, ^27^, ^28^ The apparent increase in emotional abuse in the intervention arm should therefore be interpreted cautiously. Differential reporting or variation in digital literacy across clusters may also have affected caregiver responses. These patterns are more consistent with changes in reporting behaviour than with a true increase in emotional abuse.

The absence of overall effects contrasts with Thailand's prior face-to-face pilot.^5^ Several contextual factors may have limited the ability to detect programme effects. First, the community-based and generally low-risk composition of the sample may have produced floor effects that reduced statistical power to detect change. Second, the follow-up period was limited to one month after programme completion, which may have been insufficient for sustained behavioural change to emerge. Third, variation in digital literacy and intermittent connectivity in rural areas may have influenced participant engagement with the messaging-based components of the intervention.

Despite the absence of overall effects, one specific indicator showed improvement: the average intensity score of the most challenging child behaviour (1 = “Not a problem” to 8 = “Could not be worse”) decreased significantly. This suggests caregivers acquired practical problem-solving skills, providing an early signal of tangible benefits even when broader outcomes remain unchanged.

Overall, the blended PLH delivery did not achieve measurable reductions in reported maltreatment, although it may have initiated early shifts in parental awareness and behaviour. Future iterations should focus on strengthening content on verbal conflict and emotional regulation, simplifying digital materials for low-literacy caregivers, and extending follow-up duration. Mixed-method evaluations will be important to determine whether these early changes translate into sustained behavioural improvements. These findings can be interpreted in the context of previous research. The absence of significant intervention effects in the present trial contrasts with earlier findings from a Thai face-to-face pilot^5^ and several digital programmes implemented in Asia^23^^,^^29^ These findings are also consistent with systematic reviews of parenting interventions, which report modest and context-dependent effects on harsh discipline and child maltreatment outcomes, particularly in universal community samples.^8^^,^^30^^,^^31^ This divergence may reflect two key design differences. First, the current hybrid programme was intentionally designed as a lighter-touch intervention, combining two brief in-person sessions with facilitated messaging-based support rather than the more intensive face-to-face group sessions used in earlier Thai pilots. Second, baseline levels of reported maltreatment in our sample were relatively low, which may have limited the observable scope for improvement. Together, these findings add to a growing body of evidence suggesting that brief digital or hybrid parenting support programmes may improve proximal parenting behaviours but may require longer follow-up periods or more intensive engagement to produce measurable reductions in child maltreatment outcomes.

Several features of the digital delivery format may have constrained programme effectiveness. The hybrid format's dependence on a stable internet connection proved a substantial limitation, as disruptions likely reduced participants’ engagement to the intervention and may partly explain the null findings. Furthermore, the online components limited opportunities for caregivers to practice skills prior to applying them, a key component for skills acquisition found in many in-person programmes.^7^^,^^9^

This study has several strengths and limitations. Strengths included the randomised design, assessor blinding, high follow-up rates, and culturally adapted instruments. Limitations included the short follow-up, reliance on self-reported outcomes, limited power for small effects, and connectivity constraints. In addition, the sample consisted predominantly of female caregivers (97%), which may limit the generalisability of findings to fathers or other male caregivers. The internal consistency of the Thai ICAST-TP subscale (α = 0.69) was marginally below conventional thresholds but remains acceptable for brief, adapted measures. As a universal parenting intervention, enrolment was not restricted to caregivers reporting violence or child behavioural problems at baseline; this design may have attenuated detectable intervention effects. Because participants lived in neighbouring communities, some informal information sharing between clusters cannot be excluded, which may have attenuated observable intervention effects. An additional limitation is that the modified hybrid ParentChat format was not preceded by a stand-alone pilot or feasibility study. Although adapted from existing Parenting for Lifelong Health materials and prior implementation experience in Thailand, this lighter-touch hybrid model had not previously been independently evaluated.

These findings have several implications for future research. Future evaluations should prioritise recruitment of higher-risk families, develop intervention formats that are accessible in low-connectivity settings, and test more interactive delivery approaches that support skill practice. Longer follow-up periods will also be important to explore delayed effects. Integration with qualitative data will further strengthen understanding of engagement barriers and mechanisms of change.

This trial found no short-term impacts of a blended in-person and online parenting programme on caregiver-reported maltreatment. However, the observed reduction in the severity of challenging child behaviour suggests potential early benefits. Further refinement of intervention content alongside targeting of higher risk populations may be needed before further piloting, evaluation, and large-scale implementation.

## Contributors

WP, AM, JML, FG, and PH contributed to the conceptualisation and design of the study. WP, AM, ST, JML, FG, GJMT, QH, and PH contributed to methodology development. WP, AM, ST, JML, FG, and PH contributed to investigation and project administration. AM, PH, and JML contributed to funding acquisition. AM, FG, JML, and PH contributed to supervision. WP and QH curated and analysed the data. WP, AM, JML, FG, GJMT, QH, and PH contributed to validation. AM, JML, ST, and PH contributed to resources. WP contributed to visualization. WP, AM, ST, JML, FG, GJMT, QH, and PH contributed to writing—original draft and writing—review & editing. WP and QH directly accessed and verified the underlying data reported in the study. WP and PH had final responsibility for the decision to submit for publication. All authors read and approved the final version of the manuscript.

## Data sharing statement

The de-identified dataset, Stata do-files, and full analysis log will be made publicly available on the Open Science Framework upon publication.

## Declaration of generative AI and AI-assisted technologies in the manuscript preparation process

During the preparation of this work the authors used ChatGPT (OpenAI) and Gemini (Google) in order to assist with language editing. After using these tools, the authors reviewed and edited the content as needed and take full responsibility for the content of the published article.

## Declaration of interests

AM, FG, and JML were involved in the adaptation and testing of a previous PLH programme in Thailand (PLH-YC). AM, FG, JML, and WP have received funding under the Global Parenting Initiative to promote the scale-up of PLH programmes globally. JML is the CEO of the PLH organisation, a non-profit registered in the UK, which promotes PLH programmes worldwide. These roles and funding sources were declared and managed in accordance with institutional and journal policies.

## Appendix A Supplementary data

Supplementary Fig. S1 and Tables S1–S3

Protocol_ParentChat-Thailand_PhaseII-cRCT
