# Supplementary material for: Effectiveness of a blended in-person and online parenting programme in reducing violence against children in rural Thailand: a cluster randomised controlled trial
Source: Lancet Reg Health Southeast Asia. 2026 May 28;50:100789. doi: 10.1016/j.lansea.2026.100789 (PMC13235521; doi:10.1016/j.lansea.2026.100789)
Supplement: Supplementary Fig. S1 and Tables S1–S3 [file mmc1.docx]

Contents

**Supplementary Table 1: Intervention Content and Delivery ...….….….…….…………………………….....** 2

**Supplementary Table 2: Exploratory Analysis of Positive Partner Interaction ……………………………..** 3

**Supplementary Table 3: Sensitivity and exploratory analyses for physical abuse outcomes……….………** 4

**Supplementary Figure 1a: Percentage of reporting >1 emotional abuse event ……………………………...** 5

**Supplementary Figure 1b: Percentage of reporting >1 physical abuse event ……………………………..…** 6

**Supplementary Figure 1c: Percentage of reporting >1 child abuse event ….….…………………………..…** 7

# **Supplementary Table 1: Intervention Content and Delivery.**

| Session (Week) | Core Module/Content | Delivery Method | Key Materials |
| --- | --- | --- | --- |
| 1 | Opening/Introduction  Spending one-on-one time with your child | Face-to-Face Meeting at HPH | Weekly Materials, Home Practice |
| 2 | Talking about feelings | LINE™ Group Chat, Text/Video Messages, Short Films | Weekly Materials, Home Practice |
| 3 | Keeping it positive by giving clear instructions and delivering praise | LINE™ Group Chat, Text/Video Messages, Short Films | Weekly Materials, Home Practice |
| 4 | Establishing household rules and routines | LINE™ Group Chat, Text/Video Messages, Short Films | Weekly Materials, Home Practice |
| 5 | Knowing the basics of keeping children safe from sexual violence | LINE™ Group Chat, Text/Video Messages, Short Films | Weekly Materials, Home Practice |
| 6 | Knowing online safety basics | LINE™ Group Chat, Text/Video Messages, Short Films | Weekly Materials, Home Practice |
| 7 | Positive (non-violent) discipline | LINE™ Group Chat, Text/Video Messages, Short Films | Weekly Materials, Home Practice |
| 8 | Problem-solving with children | LINE™ Group Chat, Text/Video Messages, Short Films | Weekly Materials, Home Practice |
| 9 | Closing/Celebration  Reviewing and moving forward | Face-to-Face Meeting at HPH | Not applicable |

Abbreviations: HPH = health-promoting hospital.

LINE™: LINE is a trademark of the LINE Corporation.

# **Supplementary Table 2: Exploratory Analysis of Positive Partner Interaction.**

| **Outcome** | **Group** | **Baseline** | **Post-test** | **Effect estimates** | **95% CI** | **Model type** |
| --- | --- | --- | --- | --- | --- | --- |
|  |  | **(Median, IQR)** | **(Median, IQR)** |  |  |  |
|  |  | **(n=181)** | **(n=165)** |  |  |  |
| Showing care and respect | Control | 4 (2, 7) | 5 (2, 8) | IRR 1.31 | 0.87 to 1.97 | Negative binomial |
|  | Intervention | 5 (2, 7.5) | 5 (2, 7) |  |  |  |

Abbreviations: IRR = incidence rate ratio; CI = confidence interval; IQR = interquartile range. Data are presented as Median (IQR) for positive interaction frequency at baseline and post-test. Analysis used a mixed-effects negative binomial model (IRR compares intervention vs. control at post-test).

# **Supplementary Table 3: Sensitivity and exploratory analyses for physical abuse outcomes**

| **Analysis type** | **Model specification** | **Effect estimate** | **95% CI** | **Interpretation** |
| --- | --- | --- | --- | --- |
| Primary analysis | Mixed-effects negative binomial regression (cluster and participant random intercepts; group × time interaction; adjusted for grandparent caregiver status) | IRR 0.70 | 0.30–1.62 | No statistically significant group difference |
| Sensitivity analysis (prespecified) | Mixed-effects logistic regression (any vs none; adjusted for grandparent caregiver status) | OR 0.83 | 0.25–2.82 | Results consistent with primary analysis |
| Exploratory analysis (post-hoc) | Population-averaged GEE model with log link and robust standard errors (adjusted for grandparent caregiver status) | IRR 0.46^*^ | 0.24–0.90 | Statistically significant reduction observed; interpret cautiously |

Abbreviations: IRR = incidence-rate ratio; OR = odds ratio; CI = confidence interval; GEE = generalised estimating equation.

^*^p < 0.05 (two-sided)

Primary analyses used mixed-effects negative binomial regression to account for clustering and repeated observations. Sensitivity analyses used mixed-effects logistic regression modelling the probability of reporting ≥1 incident. Exploratory analyses used population-averaged generalized estimating equations (GEE).

All models were adjusted for grandparent caregiver status (grandparent vs non-grandparent) due to baseline imbalance between study arms.

# **Supplementary Figure 1a: Percentage of reporting > 1 emotional abuse event.**

This figure presents the percentage of caregivers in the Control and Intervention groups reporting at least one event of emotional abuse at baseline, Week 4, Week 8, and Post-test (Week 14).


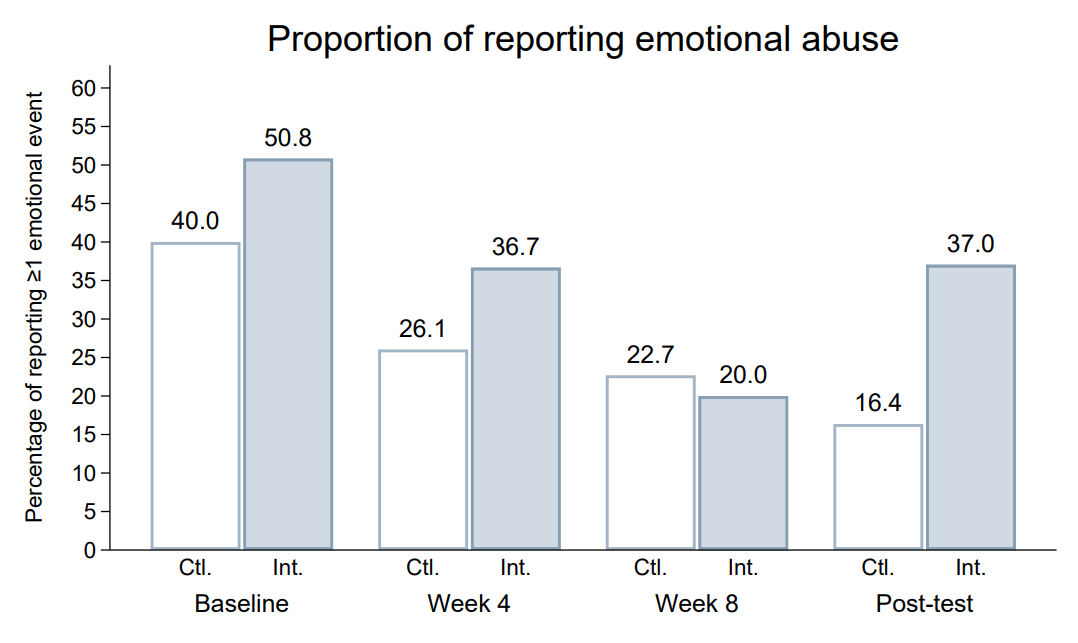


Abbreviations: Ctl., Control group; Int., Intervention group.

Post-test assessment was conducted at one-month post-intervention (Week 14). Programme duration was 9 weeks.

# **Supplementary Figure 1b: Percentage of reporting > 1 physical abuse event.**

This figure presents the percentage of caregivers in the Control and Intervention groups reporting at least one event of physical abuse at baseline, Week 4, Week 8, and Post-test (Week 14).


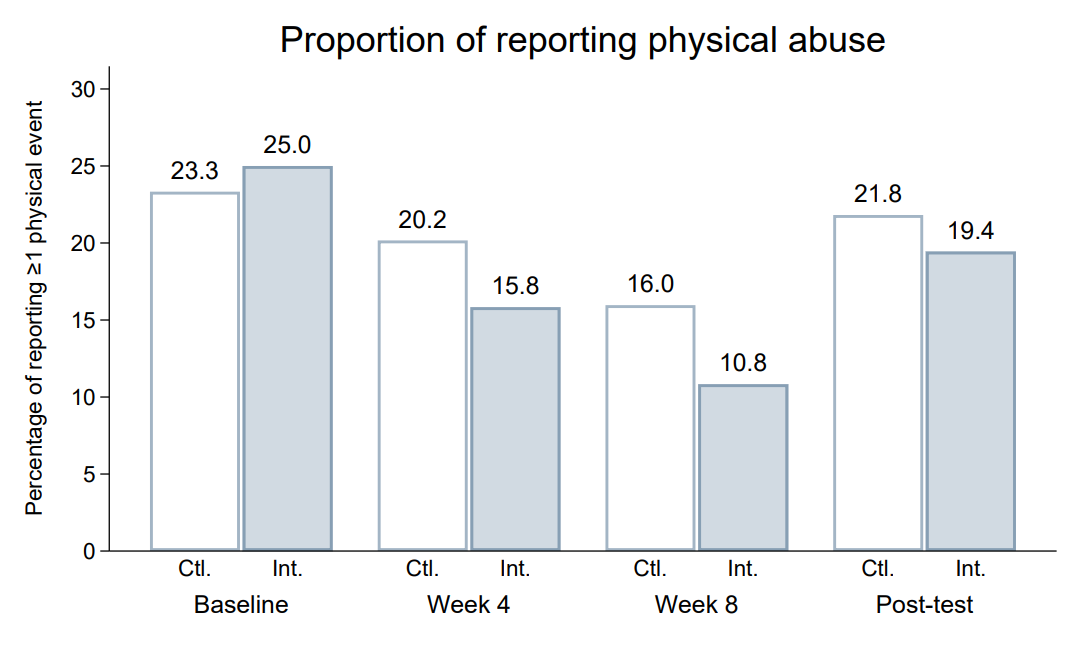


Abbreviations: Ctl., Control group; Int., Intervention group. Post-test assessment was conducted at one-month post-intervention (Week 14). Programme duration was 9 weeks.

# **Supplementary Figure 1c: Percentage of reporting > 1 child abuse event.**

This figure presents the percentage of caregivers in the Control and Intervention groups reporting at least one event of child abuse at baseline, Week 4, Week 8, and Post-test (Week 14).


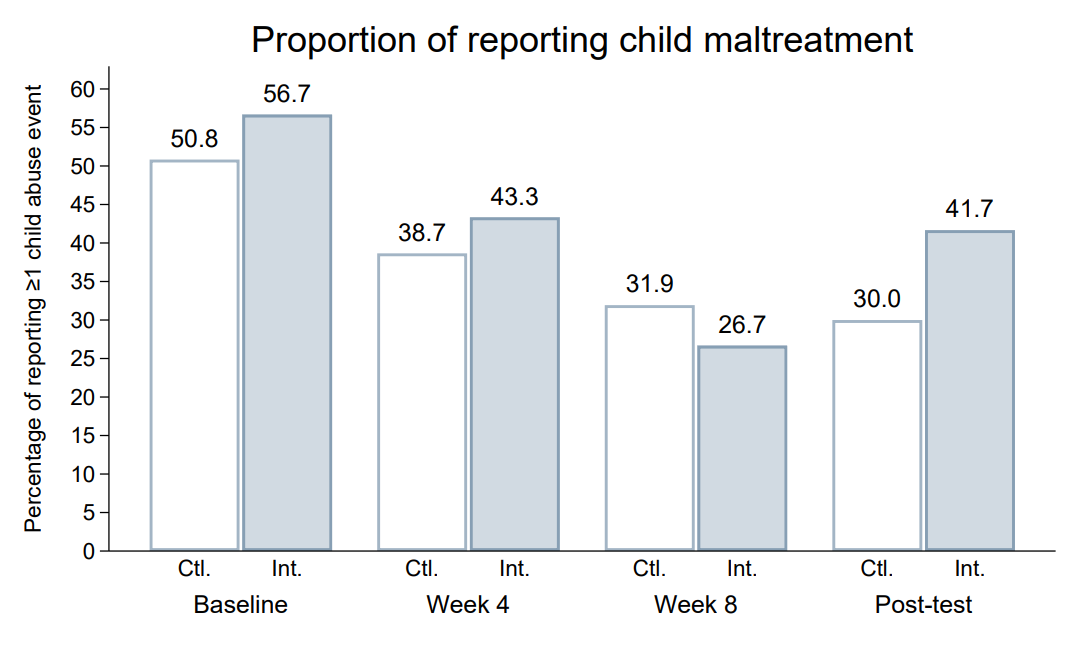


Abbreviations: Ctl., Control group; Int., Intervention group. Post-test assessment was conducted at one-month post-intervention (Week 14). Programme duration was 9 weeks.
