# Supplementary material for: Effectiveness of a blended in-person and online parenting programme in reducing violence against children in rural Thailand: a cluster randomised controlled trial
Source: Lancet Reg Health Southeast Asia. 2026 May 28;50:100789. doi: 10.1016/j.lansea.2026.100789 (PMC13235521; doi:10.1016/j.lansea.2026.100789)
Supplement: Protocol_ParentChat-Thailand_PhaseII-cRCT [file mmc2.pdf]

Study Protocol

Preliminary effectiveness and feasibility of the ParentChat online training programme to promote positive parenting and prevent violence against children in Thailand: a phase II randomised controlled trial

Table of Contents

Protocol Summary 3

Research aim 3

Objectives 3

Participants 3

Data collection and analysis 4

Full study protocol 5

A. Study team 5

B. Background 5

B.1. Literature review 5

B.2. Research aims 6

B.3. Objectives 6

B.4. Hypotheses 6

C. Evaluation 6

C.1 Study design 7

C.2 Outcome data collection 10

C.3 Outcome measurements 11

C.4 Outcome data analysis 16

C.5 Data management 18

D. Consequences of participation/ethical considerations 19

D.1 Research ethics 19

D.2 Potential of harm 21

D.3 Mitigating potential harm from the parenting modules 22

D.4 Benefits 23

References 25

Protocol Summary

Research aim

This study is a phase II randomised controlled trial (RCT) of ParentChat, an adapted online parenting support group programme for parents and caregivers with children aged 2 to 17 in Udon Thani, Thailand. ParentChat provides evidence-based parenting information and interactive learning experiences via group-based online discussions using text, audio, and visual messages. This study aims to evaluate the preliminary effectiveness of ParentChat as an intervention to reduce child maltreatment (physical abuse and emotional abuse) and improve positive parenting, parenting stress, attitudes to corporal punishment, child behaviour problems, parental mental health, parent exposure to intimate partner violence (IPV), child/adolescent exposure to risks, online safety, child learning and development, and parent support of education.

This phase II RCT will also evaluate the feasibility and acceptability of the locally adapted version by assessing the operational process, which will include implementation fidelity, programme adherence, participant satisfaction, acceptability, and feasibility.

Objectives

The objectives of the RCT are:

1. To evaluate the initial effects of the adapted ParentChat at one-month post-intervention and at the fourth and eighth weeks during programme delivery on the primary outcome of child maltreatment (physical and emotional abuse) and the proximal outcomes of positive parenting and parenting stress.
2. To evaluate at one-month post-intervention the initial effects of ParentChat on the secondary outcomes of attitudes to corporal punishment, child behaviour problems, parental mental health, and parent exposure to intimate partner violence, child/adolescent exposure to risks, online safety, child learning and development, and parent support of education.
3. To test the feasibility of the Thai ParentChat system by assessing:
    a. Implementation fidelity
    b. Programme adherence
    c. Participant satisfaction
    d. Acceptability and feasibility

Participants

Participants will include 240 parents or primary caregivers (n=120 in the intervention group, n=120 in the control group) with a child between the ages of 2 and 17 from Udon Thani, Thailand. We will also conduct a focus group with all 6 facilitators who deliver the programme and will interview 12 programme participants to assess programme acceptability and feasibility.

Data collection and analysis

Quantitative outcome data will be collected electronically at baseline and one-month post-intervention by data collectors at participants' homes. Abbreviated versions of standard, validated scales to assess the effect of the programme on the primary outcomes will be included in the survey. Computer-Assisted Self-Interviewing ('CASI') software will be used for sensitive items. If an appointment for an in-person meeting with the data collectors cannot be scheduled, participants will be interviewed telephonically. During programme delivery, programme participants will also be asked to complete a brief survey over the phone at weeks four, and eight, which will focus on primary and proximal outcomes. Data will be analysed using Stata 17. Multilevel linear regression or Poisson/negative binomial regression will be used to evaluate the intervention effects on the primary and secondary outcomes of interest.

Qualitative data will be collected via a focus group with facilitators and individual interviews with programme participants to evaluate the feasibility and acceptability of the online support group programme.

Full study protocol

A. Study team

Dr Piya Hanvoravongchai, Faculty of Medicine, Chulalongkorn University (co-PI)
Dr Amalee McCoy, Peace Culture Foundation (co-PI)
Dr Sombat Tapanya, Peace Culture Foundation (co-I)
Dr Jamie Lachman, Department of Social Policy and Intervention, Centre for Evidence Based Intervention, University of Oxford (co-I)
Professor Frances Gardner, Department of Social Policy and Intervention, Centre for Evidence Based Intervention, University of Oxford (co-I)
Ms Wilaiwan Pongpaew, Faculty of Medicine, Chulalongkorn University (co-I and research manager)

B. Background

B.1. Literature review

One billion children between the ages of 2 and 17, or 1 in 2 children worldwide, are estimated to experience violence each year [1]. The situation is no different in Thailand, where a recent survey found that 53.8% of children aged 1 to 14 had experienced violent discipline [2]. Violence against children (VAC) is a significant problem that has both short and long-term consequences on health, society, and the economy [3-12]. Adverse outcomes include poorer cognitive abilities in early childhood, increases in externalising behaviour, increases in internalising behaviour, increases in aggressive behaviour, increased behaviour problems, inattention, and attention-deficit hyperactivity disorder (ADHD), depression, smoking, heavy alcohol use, problematic drug use, sexual risk taking, and mental ill health.

Evidence-based parenting programmes that support parents in their capacity to raise children in a positive, non-violent way have been effective at reducing VAC and improving a range of child and caregiver outcomes [13-16]. An example is the Parenting for Lifelong Health (PLH) suite, a series of free, non-commercial parenting programmes developed with the aim to reduce violence in communities with limited resources. These programmes were developed and rigorously evaluated through a collaboration between the Universities of Oxford, Bangor and Reading in the United Kingdom, Stellenbosch University and the University of Cape Town in South Africa, WHO, and UNICEF. The programmes were originally developed and tested in South Africa and then adapted for other contexts, including Thailand [17]. Evidence indicates that PLH parenting programmes reduce the risk of child maltreatment [14] and increase positive parenting [13, 15]. Additionally, PLH for Teens is cost-effective in reducing the abuse of adolescents in South Africa with an estimated lifetime saving of US$2724 minimum per case [18]. In Thailand, a feasibility pilot study of an adapted PLH for Young Children (PLH-YC) programme embedded within the Thai public health system showed that the programme reduced abusive and harsh parenting [17, 19].

During the COVID-19 pandemic, there were several obstacles to implementing the in-person PLH programmes[20]. During nationwide lockdowns and health emergencies, social contact is restricted. In addition, scaling prevention programming and public health interventions is commonly limited by the sustainability of

funding resources over time for service delivery [21], particularly for in-person programmes. Consequently, the programmes have been adapted using communication technology into an online parenting support group delivered via online text messaging platforms and the contents have been adapted for young children and adolescents. This adapted programme has been called ParentChat.

This study will evaluate ParentChat when delivered to caregivers in Thailand. We will use a hybrid approach with digital technology to reach out to the parents and caregivers to promote parent-led group learning, information gathering, and capacity development to support positive parenting and improve health and well-being. The primary focus is to provide families with information on parenting that is supported by research and to encourage interactive parenting education to meet the needs of parents.

B.2. Research aims

This study aims to evaluate the preliminary effectiveness of ParentChat as an intervention for parents or caregivers with children aged 2 to 17 to reduce child maltreatment (physical abuse and emotional abuse) and improve positive parenting, parenting stress, attitudes to corporal punishment, child behaviour problems, parental mental health, parent exposure to IPV, child/adolescent exposure to risks, online safety, child learning and development, and parent support of education. This study will also assess the feasibility and acceptability of the locally adapted version by assessing the operational process, which will include implementation fidelity, programme adherence, participant satisfaction, acceptability, and feasibility.

B.3. Objectives

The objectives of this study are:

1. To evaluate the initial effects of ParentChat on the primary outcome of physical and emotional abuse, and the proximal outcomes of positive parenting and parenting stress at one-month post-intervention and at the fourth and eighth weeks during programme delivery.

2. To evaluate the initial effects of ParentChat on the secondary outcomes of attitudes to corporal punishment, child behaviour problems, parental mental health, parent exposure to intimate partner violence, child/Adolescent exposure to risks, online safety, child learning and development, and parent support of education at one-month post-intervention.

3. To test the feasibility of the Thai ParentChat system by assessing:
    a. Implementation fidelity
    b. Programme adherence
    c. Participant satisfaction
    d. Acceptability and feasibility

B.4. Hypotheses

1. Hypothesis 1: ParentChat will significantly reduce rates of child physical and emotional abuse, as measured by the ICAST-TP, in comparison to a control group.

2. Hypothesis 2: ParentChat will have a significant effect on the proximal outcomes including positive parenting and parenting stress, in comparison to a control group.
3. Hypothesis 3: ParentChat will have a significant effect on the secondary outcomes including improving positive parent involvement, attitudes to corporal punishment, child behaviour problems, parental mental health, parent exposure to IPV, child/adolescent exposure to risks, online safety, child learning and development, and parent support of education, in comparison to a control group.

C. Evaluation

The preliminary efficacy and feasibility of ParentChat will be evaluated using both quantitative and qualitative methods. Data collection will be conducted at baseline, the fourth and eighth weeks during implementation, and one month post-intervention.

The evaluation will include the following activities:

a) implementation of parenting intervention modules by local facilitators in hybrid delivery parent support groups (both in person and online);

b) pre-post and the fourth, and eighth weeks assessments during programme delivery of the impact of the intervention on reducing child maltreatment, improving positive parenting, parenting stress, and addressing risk factors associated with VAC (attitudes to corporal punishment, child behaviour problems, parental mental health parent exposure to IPV, child/adolescent exposure to risks, online safety, child learning and development, and parent support of education.

c) evaluating of implementation feasibility, programme adherence, participant satisfaction, and acceptability and feasibility.

C.1 Study design

This is a phase II RCT, observer-blinded, with two parallel groups, and designed to evaluate the preliminary effectiveness of ParentChat in reducing child physical and emotional abuse at immediate post-intervention. The study also aims to assess the feasibility and acceptability of the locally adapted version. Randomisation will be performed at the cluster level with a 1:1 allocation ratio.

Eligibility criteria

The following are the inclusion and exclusion criteria for participating parents or caregivers (n = 20 per cluster):

Inclusion criteria for parents or caregivers:

1. Primary caregiver over the age of 18 years currently caring for child between the ages of 2 to 17;
2. Has regular contact with the child and lives with the child for at least 4 nights per week;
3. Has access to a smartphone and is willing and available to join an online parenting support group to share, learn and discuss parenting; and
4. Has provided consent to participate in the study.

Exclusion criteria for parents or caregivers

1. Exhibiting severe mental health problems or acute mental disabilities
2. Already participated in the PLH-YC Thailand programme or other parenting programmes. Other parenting programmes would not include Parent Schools in Mother and Child Health clinics at public hospitals, which are provided in all provinces and districts in Thailand as 'standard care'.

The study will also assess the process of implementation.
Inclusion criteria for programme facilitators, who will deliver ParentChat, consist of the following:

1. Age 18 or older;
2. Prior completion of a ParentChat facilitator training workshop;
3. Agreement to implement the entire programme; and
4. Provision of consent to participate in the full study.

Inclusion criteria for in-depth interviews with caregivers (n = 12):

1. 4 high engagement (participate $\geq$ 6 sessions)
2. 4 low engagement (participate $\leq$ 4 sessions)
3. 4 participants who drop out before 4th session
4. Provides consent to participate in the interview

Inclusion criteria for focus groups with facilitators:

1. Age 18 or older;
2. Have delivered the entire ParentChat programme; and
3. Provision of consent to participate in the full study.

Setting

The trial will be conducted in Udon Thani, Thailand. This site was chosen as it was the pilot area for the feasibility pilot of the Thai adaptation of PLH-YC when embedded within the Thai public health system [17]. Due to this, the team has positive working relationships between senior and local government officials. Additionally, there are a number of PLH-YC programme facilitators who would be well suited to deliver the ParentChat intervention. These facilitators could be trained in a shortened three-day training course to be ParentChat facilitators. Using the existing pool of PLH facilitators would reduce the cost and time associated with recruitment and training.

Sample size

The estimated sample size was calculated using Stata 17. An exponential test comparing two independent hazard rates was employed based on the primary quantitative outcome, which was the frequency of child maltreatment in the previous month. Given type I error (α) < 0.05, power 0.8, allocation ratio 1:1, and applies the effect size in the Hazard ratio using the incidence rate ratio from the recent study of the ParentChat in six countries [22] that showed IRR = 0.57. The result shows that the minimum sample size was 108 (n = 54 in each group). When this is adjusted with an attrition rate of 10%, the minimum sample size is 120 (n = 60 in each group). However, this study uses a cluster random sampling plan, where clusters are sub-district health promotion hospitals (or health centres). To avoid bias and unreliable results, the design effect value must be additionally calculated. It was found that the design effect value is usually between 1 and approximately 5.5,

depending on the difference in size of each cluster. With clusters of similar size, the design effect value will be close to 1 [23]. Therefore, it is assumed that the design effect value equals 2 in this study. This means that the sample size will be increased twice and equal to 240.

Recruitment

This study will use a two-stage cluster sampling to recruit 240 parents or caregivers. Groups will start to operate after 20 members per group are recruited. The general recruitment strategy is described as follows:

Clusters are 12 sub-districts in Udon Thani, which will be randomly selected for the study. Some of the sub-districts that had been included in the previous study will be excluded at the stage of clusters sampling to prevent contamination bias. If there is more than one health promotion hospital in some sub-districts, only one will be selected at random. Twelve health-promoting hospitals in the selected sub-districts will be invited to partner with researchers to:

1. Provide researchers with a list of 20 randomly selected children aged 2 to 17 from each Health Promoting Hospital's database.
2. Coordinate contact with the selected children's parents/caregivers.

However, suppose the health-promoting hospitals do not allow or cannot use the randomly selected method. In that case, they can consider referring parents who could benefit from a programme that helps them manage difficult child behaviours.

Data collectors will conduct a screening interview with parents/caregivers to determine whether they meet the eligibility requirements of the study.

Allocation

The 12 sub-districts will be randomly assigned using a 1:1 allocation ratio to either the intervention or control group using a computer programme after baseline assessment, and the groups will be set up. The random sequence will be produced by an outside researcher working at the HIV Netherlands Australia Thailand Research Collaboration (HIV-NAT) who is not directly participating in the project. Following the gathering of baseline data, the project coordinator and co-investigator will contact participants via telephone to inform them of their allocation status, ensuring that participants are blinded to their status throughout the initial evaluation.

Blinding

Blinding will not be possible for those who deliver the programme due to the involvement of facilitators and coaches; additionally, participants cannot be blinded to their allocation status after the initial assessment. In order to reduce the potential of contamination, participants will not be informed of the other participants allocation status. To prevent evaluation bias, participant allocation status will be blinded from data collectors as well as statisticians contributing to data analysis. When blinding is compromised, the Research Manager will be notified as soon as possible to consult with the research team for the most appropriate course of action.

Only in cases where a participant or a member of the research team reports experiencing serious harm as a result of taking part in the study will the unblinding of participants be permitted.

Training for facilitators and coaches

The training of ParentChat facilitators will focus on the final version of the ParentChat Thailand Facilitator Manual. Trainers from the Philippines, where ParentChat has been implemented, will train the facilitators with the support of local trainers (Co-PIs Tapanya, and McCoy). Facilitators will be nurse practitioners and public health officers employed at Health Promotion Hospitals. The trainer from the Philippines will serve as a coach and will provide biweekly coaching sessions over Zoom.

The three-day training for facilitators and coaches will concentrate on developing the following abilities: 1) overview of the parenting skills and programme content; 2) managing parent groups; 3) using a collaborative approach to facilitation; and 4) managing logistics and technology. The training will take place in a hotel meeting facility or at the Boromarajonani College of Nursing, Udon Thani.

Programme delivery

The 120 parents/primary caregivers in the intervention group will be divided into six groups of 20 participants who live in the same sub-districts. Each group will be overseen by one facilitator who will deliver the programme over nine weekly sessions. The group of six facilitators will receive weekly supervision from a trained coach during programme delivery.

Content for the adapted ParentChat programme include six core contents as following: 1) spending quality time with children; 2) talking with children to understand their feelings; 3) using praise and rewards to encourage positive behaviour; 4) establishing limits through effective instruction giving and consistent household rules; 5) nonviolent discipline strategies; and 6) problem solving with children.

Core activities during sessions will be delivered through LINE, a freeware application for instant communication. The programme will provide parents with activity assignments to complete with their children, including text/audio messages, illustrated comics, and videos. The facilitators will moderate text-based discussions around the parenting theme, support parents on an individual basis, and encourage them to apply the parenting skills at home. Parents will be prompted to give feedback regarding successes and challenges applying these parenting skills via audio or text messages during the week. When the next session begins, the facilitators will give a brief voice summary of the feedback and address possible solutions to a few key challenges. During implementation, participants will be supported with the necessary technical support, including IT support. Data bundles will be provided for internet access and to incentivise engagement.

Control condition

The participants in the control group will be asked to join a LINE group in the same arrangement (20 per group) as those intervention group but they will not be any content or have any activity. There will be only general conversation among the group members. At the time of the intervention, the control will receive

standard care but not any active interventions. 'Standard care' includes access to Parent Schools in Mother and Child Health clinics at public hospitals, which are provided in all provinces and districts in Thailand.

C.2 Outcome data collection

Data collectors with experience working at the community level with low-income families will be recruited and trained extensively by the study team on ethics, informed consent, and data collection procedures. A detailed protocol will guide data collection to ensure consistency across data collectors. Surveys will be translated into Thai by the researchers and back-translated by an independent translator to ensure accuracy.

Data collection during the trial will take place at four time points. In addition to baseline and one-month post-intervention assessment, which will be done in person, assessments during programme delivery will be conducted telephonically during weeks four, and eight. These assessments will assess changes in target outcomes over the past two weeks.

During pre-post assessments, data collectors will visit participants' homes to complete surveys with them on tablets. Data collectors will sit beside participants and go through the survey together. To reduce the potential for error, data collectors will enter participants' responses into the tablet while showing them the screen. For sensitive sections of the questionnaire (i.e., those relating to IPV and physical/emotional abuse), the tablet is given to the parent, who is able to complete it on their own, using earphones to listen to the questions by audio if they have difficulties with literacy. If parents would prefer to continue completing the survey with the data collector for the sensitive items (as opposed to using CASI), they will continue to use this approach.

C.3 Outcome and process evaluation measurements

Summary of outcomes and process evaluation measurements are shown in Table 1. All measures will be translated into Thai language and back translated to check the accuracy of the translation.

Sociodemographic information

Basic demographic questions about the parent/caregiver and the child will be asked using 25 items. Twenty three of these items are from those used in evaluations of PLH-YC in Thailand [17]. These items include 1) parent/caregiver variables (eleven items): age, sex, gender, language, ethnicity, citizenship, religion, marital status, education level, basic literacy, and employment; 2) Child variables (three items): age, sex, school enrolment; 3) Child relationship variables (three item): the child's relationship to the caregiver; 4) household variables (six items): total monthly household income, other caregivers in the household, number of children in their care, biological parents, number of other adults in the household, and number of other children in the household. In addition, two items adapted from the Washington Group questions [24] on parent/caregiver and child disability will be asked whether they have any difficulties and, if so, what type of difficulty they have.

Primary outcome

1. *Child maltreatment – physical abuse and emotional abuse*

Four items adapted from a reduced version of the International Society for the Prevention of Child Abuse and Neglect (ISPCAN) Child Abuse Screening Tool-Trial Parent version (ICAST-TP) [25] will be used to determine physical abuse and emotional abuse. The evaluation will be repeated four times consisting of the evaluation at baseline, post-test, and every four weeks during programme delivery. This study will evaluate the incidence of child abuse both in overall abuse by combining all subscales of two items assessing physical abuse and two items assessing emotional abuse. Participants report on the incidence of violent discipline use over the past four weeks using a frequency score of 0 to 8 or more times (e.g., how often did you discipline by spanking, slapping, or hitting with their hand over the past 4 weeks.).

Proximal outcomes

2. Positive parenting

To evaluate positive parenting, five items adapted from the Alabama Parenting Questionnaire (APQ) [26, 27] will be utilised at baseline, and post-test. We will also ask two of these questions at the fourth and eighth weeks during programme delivery for a total of four data collection points. Parents/caregivers will report on their behaviour from the previous two weeks. The two questions are, "how many times in the past 2 weeks did you play games or do other fun things with your child?" and "how many times in the past 2 weeks did you praise your child if he/she behaves well?" The items will be evaluated on a scale of 0 to 7, or 8 or more times, for frequency.

3. Parenting stress

To evaluate parenting stress, five items adapted from the Parental Stress Scale (PSS) [28] will be utilised at baseline, and post-test. We will also ask one of these questions at the fourth and eighth weeks during programme delivery for a total of four data collection points. The question asked about whether they disagree or agree with the statement, "The major source of stress in my life is my child(ren)." The items will be evaluated on a 5-point Likert scale of 0 to 4 (0 = strongly disagree; 4 = strongly agree).

Secondary outcomes

4. Attitude toward corporal punishment

One item from the UNICEF Multiple Indicator Cluster Surveys (MICS): Child Discipline module [29] will be utilised to evaluate attitudes towards corporal punishment at baseline and post-test. Parents/caregivers will be asked whether they disagree or agree with the statement "In order to bring up, raise up, or educate a child properly, the child needs to be physically punished". Parents will report their attitude using a 5-point Likert scale of 0 to 4 (0 = strongly disagree; 4 = strongly agree).

5. Child behaviour problems

The Child and Adolescent Behaviour Inventory (CABI) [30] will be used to measured child behaviour problems, using a total of 14 items. The behaviour of the child over the previous two weeks will be reported by the parents/caregivers (0 = Not True; 2 = Very True). Problem behaviours will be assessed using the irritability subscale (4 items, for example, "has frequent mood changes") and the externalising subscale (10 items, for example, "often lies or cheats"). The items on both subscales are summed to produce a final score for child

behaviour problems. Additionally, two items modified from the Three Problem Scale-Parent Report [31] will be utilised to assess whether the specific concerns or issues change during an intervention. Parents will report whether the child's behaviour problems have occurred in the past two weeks and select the most problematic behaviour from a list of 10 and indicate how challenging the behaviour was on a scale of 0 to 8+ (0 = not a problem, 8+ = could not be worse).

6. Parental mental health

Parental mental health will be evaluated using the depression subscale of the Depression Anxiety Stress Scale 21 (DASS-21) [32]. There are seven items in the depression subscale, such as " I felt down-hearted and sad." Parents will report their incidence of depressive symptoms during the past two weeks using a Likert scale (0 = Never; 3 = Always).

7. Parent exposure to IPV and intimate partner coercion

Utilising seven items from an adapted version of the WHO Violence Against Women Instrument (VAWI), which are part of the WHO Multi-Country Study Questionnaire on Women's Health and Life and Domestic Violence against Women [33], adults' self-reports of IPV and coercion over the past week will be evaluated. We separate the questions for men and women and ask them about experiencing and inflicting violence in compliance with WHO ethical and safety standards for intervention research on violence against women. Questions for men and women are segregated in accordance with WHO ethical and safety recommendations for intervention research on violence against women [34], and both will be asked about experiencing and perpetrating.

As a result, the questions are divided into two sections, the first consisting of five items and the second consisting of two items. Women will be questioned about their experiences as victims of violence in the first section, while men will be questioned about their experiences as perpetrators of violence. The items in this section include frequency of being respected ("my partner [I] respected my [my partner's] feelings in disagreement"), emotional violence ("my partner [I] insisted on knowing where I [my partner] was at all times"), financial autonomy restriction ("my partner [I] refused to give me [my partner] money for household expenses, even when my partner [I] had money for other things."), physical assault ("my partner [I] pushed, shoved, or slapped me [my partner]"), and psychological aggression ("my partner [I] insulted, shouted, yelled, or swore at me [my partner]"). The roles of men and women are reversed in the second section, with women being questioned about perpetrating acts of violence while men are the victims. The items in this section include the frequency of defending violence ("at a time that my partner [I] hit me [my partner], I [my partner] fight back physically") and partner mistreatment ("I [my partner] physically mistreated my partner [me] when my partner [I] was not physically mistreating me [my partner]"). All answers are coded on a frequency scale of 0 to 7, or 8 or more times. Please note that the squared brackets in the question examples refer to questions being asked to men.

8. Child/adolescent exposure to risks

Child/adolescent exposure to risks will be evaluated using seven items related to exposure to risky situations. These items have been adapted from those used in evaluations of ParentApp for Teens in Tanzania[35] and

ParentText in South Africa. The items ask about how many times their child has been in any of these situations in the past month. For example, "they were out with friends and ended up walking home alone in the dark", and "they went to buy food at the market or shop alone at night". All answers are coded on a frequency scale of 0 to 7, or 8 or more times.

9. Online safety

Online safety will be evaluated using two items adapted from the Global Kids Online: hurtful and bullying behaviour subscale [36]. One item asks, "how many times has your child been gone to meet someone face-to-face that they first got to know on the phone or internet in the past month?" And "how many times has your child brought items home when you didn't know how they got them?" All answers are coded on a frequency scale of 0 to 7, or 8 or more times.

10. Child learning and development

Child learning and development will be evaluated using six items from the UNICEF Multiple Indicator Cluster Survey (MICS) early childhood development [37]. The items ask about how frequently parents/caregivers spend one on one time with their child, such as "how often in the past week did you read books to or look at picture books with your child?" and "how often in the past week did you tell stories to your child?". The items will be evaluated on a scale of 0 to 7, or 8 or more times, for frequency. These items will be asked up to age 9.

11. Parent support of education

Parent support of education will be evaluated using five items assessing parent involvement and support for school. These items are based on those used within the Latino Family Study [38]. The items ask about how frequently in the last month the following has happened towards their child, such as "how frequently in the last month you talked about the importance of school and education with your child?" and "how frequently in the last month have you praised your child for working hard at school?". The items will be evaluated on a 5-point Likert scale of 0 to 4 (0 = Never, 1 = Almost never, 2 = Sometimes, 3 = Often, and 4 = Always).

Process evaluation outcomes

1. Implementation fidelity

Self-report checklists by facilitators will be used to assess programme implementation fidelity. These checklists include specific activities for each session, such as home practice discussion and coverage of core content. Following that, a ratio of programme implementation to programme design will be created for the self-report scores [39]. According to Borrelli and colleagues, "high treatment fidelity" is defined as a criterion of 80% programme fidelity [40].

2. Programme adherence

The rates of enrolment, attendance, and dropout will be examined to assess programme adherence. The percentage of parents that participate in the programme will determine enrolment rates. For each ParentChat chat session, parents are considered to have participated if they sent at least one message. The ratio of

sessions attended to total sessions in the programme will be used to calculate the average attendance rates for enrolled participants. The percentage of enrolled participants who fail to attend for at least two consecutive sessions and then fail to attend for any sessions at a later stage will be referred to as the dropout rate.

3. Participant satisfaction

In the post-test assessment, parents and caregivers attending at least one programme session will be asked about their satisfaction with the intervention using five items from a satisfaction questionnaire used in evaluations of PLH-YC in Thailand. Overall participant satisfaction will be calculated in the form of mean and standard deviation values (1 = Very unhelpful; 5 = Very helpful).

4. Facilitator and participant engagement

The level of engagement will be determined by the number, type, and word count of messages that facilitators and participants send to the online groups during each session. Additionally, the number of activities completed by each participant (such as home assignments) will be used as a measure. Message logs will be exported into a secure database and pseudo-anonymised messages prior to sharing with local research team. The researcher will conduct a content or narrative analysis of text messages to evaluate the acceptability of programme content, quality of programme delivery, and interactions and communication patterns between facilitators and participants. A summary of the challenges or issues found regarding participant and facilitator engagement will be included in the weekly assessment.

5. Acceptability and feasibility

After the programme, a focus group discussion (FGD) with the facilitators (n = 6) and individual interviews with programme participants (n = 12) will be conducted to assess programme acceptability and feasibility. The inclusion criterion for participation in individual interviews will be attendance at least one session. Participants will be purposefully selected using the following criteria: high engagement (n = 4), low engagement (n = 4), and dropouts (n = 4). Additionally, the participant's sex will be taken into consideration in the selection criteria. Four to six fathers or male caregivers will be invited to participate in the individual interviews for the male engagement. All parent interviews will be conducted by trained researchers. The participant interviews will be conducted in person in the participants’ homes for about 60 minutes, or they may be conducted over the phone if the participants are unable to do in person interviews. The interview will be audio recorded, and notes will also be taken in case the recording doesn't work. The FGD will be both audio and video recorded, and written notes will be taken as back up. The FGD will take between 60 and 90 minutes and will be conducted in person at the health promoting hospital or at the Boromarajonani College of Nursing, Udon Thani.

The focus group and interviews will be conducted during the post-evaluation with the following themes:

- Participants’ observed change in parent-child relationships at home during programme;
- Cultural acceptability and appropriateness of programme delivery, and key programme components; and

- Existing barriers to participation during sessions and engagement in home practice and other activities.

The FGD will also focus on the benefits to the programme (in terms of content, logistics, and process), problems faced by facilitators during programme delivering on both a logistical level (such as recruiting, session length, and online delivery format) and process (such as using a collaborative approach and/or explaining concepts such as child-led play).

Following the feedback on acceptability and feasibility expressed by participants, all interviews and FGD records will be transcribed using computer programmes such as NVivo to organise the information. Thematic analysis [41] will be used to analyse the data. The researchers will evaluate and code the transcripts. The codes will be defined into the following themes: 1) overall experience; 2) cultural and contextual relevance of programme themes and skills, structure, schedule, and logistics; 4) barriers and facilitators of programme participation, engagement, and implementation; and 5) possibility of programme scale-up. This is also looking for which themes emerge from the data. The validity and representativeness of each theme will be emphasised during the research team's discussion of the findings. Lastly, we will choose a few data extracts that highlight important themes and opposing perspectives.

Table 1. Summary of outcome and process evaluation measurements

Outcome	Study Measurements	Timing	Items
**Demographic factors**			
Parent/primary caregiver, child and family demographics	General demographics questions; PLH-YC in Thailand[17]	Baseline	23
	Disability; Washington Group questions [24] (adapted)	Baseline	2
**Child maltreatment**			
Child maltreatment: physical abuse and emotional abuse	ISPCAN Child Abuse Screening Tool-Intervention – Physical and Emotional Abuse Subscales [25]	Baseline, the fourth and eighth weeks, and Post-test	4
**Proximal outcomes**			
Positive parenting and positive parent involvement	Alabama Parenting Questionnaire (APQ) [26, 27]	Baseline, the fourth and eighth weeks, and Post-test	2
		Baseline & Post-test	3
Parenting stress	Parental Stress Scale (PSS) [28]	Baseline, the fourth and eighth weeks, and Post-test	1
		Baseline & Post-test	4
**Secondary outcomes**			
Attitudes to corporal punishment	Multiple Indicator Cluster Survey [29]: child discipline module	Baseline & Post-test	1

Outcome	Study Measurements	Timing	Items
Child behaviour problems	Child and Adolescent Behaviour Inventory (CABI) [30]<br><br>Three Problem Rating Scale [31]	Baseline & Post-test	14<br><br>2
Parental mental health	Depression Anxiety and Stress Scale 21 (DASS-21) [32]: depression subscale	Baseline & Post-test	7
Parent exposure to IPV and intimate partner coercion	WHO Multi-Country Questionnaire on Women's Health and Domestic Violence against Women (WHO) [33]	Baseline & Post-test	7
Child/Adolescent exposure to risks	Adapted from those used in evaluations of ParentApp for Teens in Tanzania and ParentText in South Africa	Baseline & Post-test	7
Online safety	Global Kids Online - Hurtful and bullying behaviour subscale [36]	Baseline & Post-test	2
Child learning and development	Multiple Indicator Cluster Survey (MICS) [29]: early childhood development	Baseline & Post-test	6
Parent support of education	Adapted from those developed for Rosie Ceballo's Latino Family [38]	Baseline & Post-test	5
**Process evaluation outcomes**			
Implementation fidelity	Facilitator checklists	Post-test	n/a
Programme adherence	Attendance registration	Post-test	n/a
Participant satisfaction	Parent/primary caregiver overall satisfaction questionnaire subscale of those used in evaluations of PLH-YC in Thailand	Post-test	5
Facilitator and participant engagement	Process monitoring reports	Post-test	n/a
Acceptability and feasibility	Qualitative interviews	Post-test	n/a
		**Total items at baseline**	**90**
		**Total items at the fourth and eighth weeks**	**7**
		**Total items at post-test**	**70**

C.4 Outcome data analysis

Stata 17 will be used for the analysis of general demographic data and the effectiveness of the programme as follows:

Descriptive statistics will be employed to analyse the general demographic data. The average and standard deviation will be used for the continuous data unless that data obviously violates a normal distribution. If the data is obviously a non-normal distribution, the median and interquartile ranges will be used. In addition, frequency and percentage values will be utilised for the categorical data.

All intervention effects will be evaluated utilising an intention-to-treat design, that includes all participants recruited in the study regardless of whether they complete the programme or all the assessment points [42]. To handle missing data, the imputation or deletion method might not be suitable due to the limited sample size. We plan to manage this problem as follows:

1) For general demographic information, there is only a measurement time point. Once the data is collected by facilitators before programme delivery, the data will be checked immediately for completion. If any part of the information is found to be incomplete, we will try our best to contact the participants through various channels to request as much information as possible during the period that the participants remain in the programme.
2) The missing data in the primary or secondary outcomes will be carried out in the same way as the general demographic. The data will be checked immediately at each time point of the data collection. If any part of the information is found missing, it needs to be followed up on during the time that the participants are still in the project. In the event that the missing data occurs because participants drop out, we will try to contact the participants to find out whether their dropout is related to any factors in the project. If it is evident that there are conflicts or other project-related reasons leading to dropouts, the issues will be addressed to resolve them. If the dropout occurs randomly or it is just a personal reason, the project will go on, and the data will be followed up as much as possible. However, if there are data from at least two time points in each outcome, it is adequate for longitudinal data analysis using multilevel methods, and the missing data does not need to be imputation.

To evaluate the hypothesis regarding measuring effectiveness on primary and secondary outcomes, two-tailed tests given a type I error of p $(\alpha) < 0.05$ and power = 0.8 will be used. Along with the estimated values, 95% confidence intervals and p-values will be presented. Multilevel analysis (MLA) was used to analyse the results because the data is multilevel structured. The data from repeated measurements taken at different times makes up the lowest level. The second level is the individual level, and the third level is the level of sub-district. Primary and secondary outcomes that are on a frequency scale, including child maltreatment, positive parenting, parent exposure to IPV, child/adolescent exposure to risks, online safety, and child learning and development, which are discrete data, will be utilise distribution testes to determine whether to use a Poisson regression or a negative binomial regression if the data is overdispersion. For the ordinal scale outcomes, including parenting stress, attitudes toward corporal punishment, child behaviour problems, parental mental health, and parent support for education, we will use a linear regression to measure the differences between the two groups. A two-level model will be performed comparing post-intervention effects across all outcomes, controlling for baseline and the random effect will be the cluster, and participants ID. In addition, a three-level model will be performed comparing time trends across child maltreatment and proximal outcomes.

C.5 Data management

Participants will consent to the storage of their data, and they will be informed of who the involved members are, and that the data will be made available openly when the study is finalised.

Data collectors will be trained to standardise procedures, and a manual will be provided for them with detailed instructions and contacts to carry out the data collection throughout the research project. Data will be collected through the Open Data Kit using tablets while offline.

A lead data collector will review the collected data before it is primarily submitted to IDEMS International's ODK Central server until the data collections will be completed. After that, the data will be back-ups to the severs at Oxford University. The servers run incremental back-ups daily on two different servers within the university. Immediately after an online survey is submitted, all data collected will be encrypted and saved on the servers, which are accessible only to the research members. Firewalls and regular data backups are part of the robust safety system. Transmission will take place daily. However, the systems will be tested for the first time, and the data will also be paper copies as backups.

To protect participant confidentiality, all data will be anonymised before being analysed. All transcriptions and audio recordings of in-person and online focus groups will be de-identified at the earliest opportunity. To ensure confidentiality, data will be pseudo-anonymised. Data will only be accessed by the research team, managed by the research manager. All electronic data such as transcripts of qualitative data, will be password-protected and accessed on a need-to-know basis. Paper copies as back-ups or any physical copy will be stored at a specified secure location (locked) with a designated responsible person to look after them. Data will be secured against confidentiality violations.

Data cleaning will follow standard procedures across datasets and timepoints. A data monitoring process will be implemented to check the quality of the data as the project collects it and to correct any mistakes in a timely manner. Data will be cleaned at the end of all data collection stages. The final merged dataset will be kept separately from the individual baseline and post-test assessment datasets, so that data reference points are accessible during the data validation process.

At the end of the study, any documents containing personally identifiable information will be destroyed.

After each participating family has given their informed consent, pseudonymised data for each will be linked using a unique study number provided to them. This will be done to comply with any study withdrawals given by the participants during the study. At the end of the study, the link between the unique study number and the individual identifier will be destroyed. Pseudonymised data identifiers will be stored separately from research data and linked through a unique study number. Linked information is never linked to the final dataset, which is completely anonymised; instead, it is kept in password-protected laptops and files.

After the conclusion of this study, individual identifiable data will be deleted immediately. All electronic identifiable data will be deleted from external hard drives.

<u>D. Consequences of participation/ethical considerations</u>

D.1 Research ethics

Ethical approval

Ethical approval will be obtained from the Institutional Review Board, Faculty of Medicine, Chulalongkorn University (Med Chula IRB), prior to the study's start.

Informed consent procedures for parents/primary caregivers

Informed consent procedures will be carried out by trained data collectors in participants' homes or community centres or over the phone if an in-person meeting is not feasible. If there are limitations to participants' understanding of the methodology and aim of the study, a participant will be provided with an oral consent script that contains all the information about the study's design.

Giving a detailed verbal description of the study is part of the informed consent process. Participants will be reminded that participation is voluntary and that they can refuse to participate in either the intervention or research and/or can discontinue participation at any time without penalty. In order to protect participants' privacy, staff will also outline the ground rules for the online communities. Participants will be made aware that all information shared in the online groups will only be used for research and training purposes.

The methods for protecting participant confidentiality will be explained to participants, including the use of non-personally identifying ID numbers instead of names on research materials and the storage of electronic data on servers that are password- and encryption-protected. Reminders will be given to participants so that they can access information on social or health services without participating in the study. All participants will receive the investigators' phone numbers and informed that they can call to ask any questions they may have regarding the study or their rights as human subjects. Participants may consent to participate only after having the study information explained to them and there has been an opportunity for questions. The research team will assure that they are fully informed about the study and have had the chance to ask any questions that might have arisen.

Participants will have an opportunity to determine whether to consent immediately or within one week. If the participant consents, the Participant Consent Form will be signed by both the participant and the interviewer on the same date before the first point of data collection starts within this one-week period. An oral consent approach will be used if consent procedures are conducted over the phone.

Informed consent procedures for facilitators

Prior to the study, researchers will provide participants with a written information sheet. Facilitators may consent to participate only after receiving a written copy of the information sheet and having an opportunity to ask questions prior to the FGD. The researcher will ensure that facilitators have had an opportunity to ask any questions and are properly informed about the study. Facilitators will have an opportunity to determine whether to consent immediately or within one week. Facilitators will be asked to email a signed consent form to the researcher as their sign-off. An oral consent approach will be used for facilitators who do not have access to email. The method will be comparable to the informed consent process for parents or caregivers that was previously detailed.

Confidentiality

LINE will be used as a social media messaging platform to conduct parenting support groups.

Due to the nature of group interactions, like focus groups in any research, ParentChat groups will constitute an exception to full confidentiality for participants. Although there will be "Ground Rules" for participation in the ParentChat groups, including respect and confidentiality, the nature of group-based interaction implies that participant responses in the group chats will not only be seen by the research team. Before joining a ParentChat group, participants will go through an informed consent process where they will be made aware of this limitation. Data confidentiality in the research process will be an exception when there is an ethical need to protect participants from harm to themselves or others.

All of the appropriate steps will be taken to ensure each participant's confidentiality. To ensure that participants' personal names and the names of their children are kept private, each participant will be given a research identification number. Weekly exports of group chat content and engagement data will be made using password-protected hardware and password-protected files kept on secure servers. Only the co-principal investigators and the investigator will be allowed access to these password-protected, secured, and encrypted systems. Prior to any data analysis, all assessment data will be anonymised.

Other ethical issues

Completing lengthy surveys may possibly strain research participants and have a negative impact on the reliability of self-report data [43]. Therefore, we have tried to keep the surveys as short as possible, and we estimate that the pre-post questionnaire will take about 30 minutes to complete. Using CASI techniques can also increase data collecting effectiveness and efficiency while lowering respondent burden. It has been demonstrated that the CASI technology is very acceptable to research assistants and participants, that it speeds up data collection, and that it is more accurate than paper questionnaires [44].

D.2 Potential of harm

This study's main objective is to assess the effectiveness of an online parent support group programme. We feel that any potential harm to participants will be outweighed by the potential benefits of the research and intervention in lowering the risk of child abuse. However, we must take consider two different types of potential harm: participating participation in the study and participation in the intervention.

Potential harm from the study

It is possible that giving respondents the chance to talk about parenting and parent-child relationships during assessments will cause them distress [45]. Researchers with considerable expertise conducting research with at-risk families will be on the study team, and they will be able to discuss or supervise any difficulties that participants may have after completing the surveys. Referrals will be provided if a participant needs to obtain more extensive support, such as consulting with a counsellor or visiting a clinic. The research team will prepare a list of contacts for referrals to local organisations, which will be provided to all participants at the time of study recruitment.

All research staff will receive training on ethical practices and protocols for work involving human subjects. All participants will be informed during the consent stage that confidentiality will be maintained as much as possible in online groups unless it becomes apparent that they are at risk of significant harm or of threatening anyone else. Participants will also be informed that they are not required to answer any questions with which they are uncomfortable and that they are able to stop at any time without any negative consequences.

Disclosure of harm

Participant disclosure regarding harsh parenting may indicate that significant harm has occurred or is at high risk of occurring. Researchers have a responsibility to protect children who could be vulnerable to significant harm due to abuse, neglect, or any other risk. It should be underlined that helping children whom the research identifies as being in need is an ethical responsibility.

The protocol listed below will subsequently be followed to minimise any potential harm to children or adults that might occur throughout the study:

1. If information is disclosed that suggests that any member of the household is experiencing harm or at risk of significant harm, the researcher will discuss concerns with the respondent at the end of the interview; if there is reasonable concern, the PI and research manager will be informed for further guidance;

2. If the household member experiencing harm or risk of harm is a child, the researcher will discuss with the parent the possibilities for referral to child welfare, health organisations, and other services;

3. If the harm or risk of harm is considered to be significant, the research staff will inform local child protection services via the local One Stop Crisis Centre or the Ministry of Social Development and Human Security hotline 1300;

4. If severe abuse is disclosed in data collection, children will be immediately referred to social or medical services and the participant will be automatically excluded from the study;

5. If the decision is made to act, the participant(s) will be informed and referral will be made (*Please see attached child protection and referral form)*;

6. All staff will also receive additional training from the research team on how to respond to these situations in alignment with the study's referral protocols;

7. Weekly supervision meetings with all project staff will allow discussion of issues that arise concerning harm or risk of harm to research subjects and children;

8. Finally, if we determine that respondents or their families have experienced significant harm as a result of participation in the research study (i.e., severe abuse, suicidality, intimate partner violence, or other potential psychological or physical injuries), we will cease further activities until these issues can be addressed adequately.

D.3 Mitigating potential harm from the parenting modules

We have considered the potential risk of harm from participating in the intervention and will be monitoring this throughout the project [46]. There may be potential psychological harm caused by participation in the parenting programme as a result of bringing up difficult experiences in caregivers' own childhoods or confronting IPV at home. Due to a participant's partner disapproving of participating in the study or programme, there may also be the risk of experiencing intimate partner conflict. Although this has rarely occurred in in-person research on parenting programmes in similar settings, it will be monitored as a potential adverse event. The research team will examine these occurrences and make appropriate reports and referrals. Nevertheless, decades of study on parenting strategies, including numerous randomised trials in LMICs [47], have not identified any evidence that these interventions are harmful. Instead, there is significant evidence that they benefit both children and parents, and have high parent satisfaction. As a result, this study will focus on the potential benefits and risks of parenting programmes for both parents and children. Specifically, we have included a safeguarding measure to minimise potential risk of harm and prevent any unintended consequences from including questionnaire items on IPV by ensuring men and women from the same household are not both interviewed about IPV. This approach complies with the WHO research guidelines on violence against women [48] as well as the ethical and safety recommendations for such studies [34]. To protect female participants, men will not be asked the IPV questions if they state that their spouse is also a participant in the project.

Programme participation will be completely voluntary, with penalties for refusing to participate. In addition, numerous other trials, including a number of evaluations in other low-resource contexts, show no evidence of harm from discontinuing parenting interventions [47]. Lastly, the programme will not continue implementation until any harm has been effectively addressed; moreover, the programme will be modified if there is any indication of significant harm from either intervention condition at the post-test.

D.4 Benefits

To promote programme completion and support internet access during the implementation period, parents/primary caregivers will receive 125 Thai Baht (approximately 2.88 GBP) and 150 Thai Baht (approximately 3.46 GBP) for completing the baseline and post-intervention questionnaires, respectively. During programme delivery, parents/primary caregivers will also receive 25 Thai Baht (approximately 0.58 GBP) for completing at weeks four, and eight. For the in-person session of ParentChat, participants will be reimbursed for transportation costs and provided with refreshments.

For the process evaluation, we will provide each of the 12 parents/primary caregivers who complete the post-interview with 100 Thai Baht (approximately 2.30 GBP). No separate compensation will be provided to participants for completing the satisfaction questionnaire.

ParentChat facilitators will be provided with a daily fee of 800 Thai Baht (approximately 18.43 GBP) for their services. The coach will be provided with 2,000 Thai Baht per coaching session (approximately 46.09 GBP) following the first seven parenting programme sessions. For participation in the focus group discussion, each facilitator will receive 300 Thai Baht (approximately 6.92 GBP).

References

1. Hillis S, Mercy J, Amobi A, Kress H. Global prevalence of past-year violence against children: A systematic review and minimum estimates. Pediatrics. 2016;137(3):e20154079.

2. National satatistical office. Thailand Multiple Indicator Cluster Survey 2022. 2023.

3. Heilmann A, Mehay A, Watt RG, Kelly Y, Durrant JE, van Turnhout J, et al. Physical punishment and child outcomes: a narrative review of prospective studies. Lancet. 2021;398(10297):355-64.

4. Cuartas J, Weissman DG, Sheridan MA, Lengua L, McLaughlin KA. Corporal Punishment and Elevated Neural Response to Threat in Children. Child Development. 2021;92(3):821-32.

5. Kirsch DE, Tretyak V, Radpour S, Weber WA, Nemeroff CB, Fromme K, et al. Childhood maltreatment, prefrontal-paralimbic gray matter volume, and substance use in young adults and interactions with risk for bipolar disorder. Scientific Reports. 2021;11(1):123.

6. Hughes K, Bellis MA, Hardcastle KA, Sethi D, Butchart A, Mikton C, et al. The effect of multiple adverse childhood experiences on health: a systematic review and meta-analysis. Lancet Public Health. 2017;2(8):e356-e66.

7. Tan M, Mao P. Type and dose-response effect of adverse childhood experiences in predicting depression: A systematic review and meta-analysis. Child Abuse Negl. 2023;139:106091.

8. Strathearn L, Giannotti M, Mills R, Kisely S, Najman J, Abajobir A. Long-term Cognitive, Psychological, and Health Outcomes Associated With Child Abuse and Neglect. Pediatrics. 2020;146(4):e20200438.

9. Martin A, Muñoz JM, Braza P, Ruiz-Ortiz R, Del Puerto-Golzarri N, Pascual-Sagastizábal E, et al. Parental Corporal Punishment and Peer Victimization in Middle Childhood: A Sex-Moderated Mediation Model of Aggression. Front Psychol. 2020;11:573329.

10. Fulu E, Miedema S, Roselli T, McCook S, Chan KL, Haardörfer R, et al. Pathways between childhood trauma, intimate partner violence, and harsh parenting: findings from the UN Multi-country Study on Men and Violence in Asia and the Pacific. The Lancet Global Health. 2017;5(5):e512-e22.

11. Widom C. Long-Term Impact of Childhood Abuse and Neglect on Crime and Violence. Clinical Psychology: Science and Practice. 2017;24.

12. Fry D, McCoy A, Swales D. The Consequences of Maltreatment on Children’s Lives: A Systematic Review of Data From the East Asia and Pacific Region. TRAUMA, VIOLENCE, & ABUSE. 2012;13(4):209-33.

13. Ward CL, Wessels IM, Lachman JM, Hutchings J, Cluver LD, Kassanjee R, et al. Parenting for Lifelong Health for Young Children: a randomized controlled trial of a parenting program in South Africa to prevent harsh parenting and child conduct problems. J Child Psychol Psychiatry. 2020;61(4):503-12.

14. Cluver LD, Meinck F, Steinert JI, Shenderovich Y, Doubt J, Herrero Romero R, et al. Parenting for Lifelong Health: a pragmatic cluster randomised controlled trial of a non-commercialised parenting programme for adolescents and their families in South Africa. BMJ Glob Health. 2018;3(1):e000539.

15. Lachman JM, Cluver L, Ward CL, Hutchings J, Mlotshwa S, Wessels I, et al. Randomized controlled trial of a parenting program to reduce the risk of child maltreatment in South Africa. Child Abuse Negl. 2017;72:338-51.

16. Morello L, Caputi M, Scaini S, Forresi B. Parenting Programs to Reduce Recurrence of Child Maltreatment in the Family Environment: A Systematic Review. Int J Environ Res Public Health [Internet].

2022 Oct 14 [cited 2023 15 March]; 19(20). Available from: https://mdpi-res.com/d_attachment/ijerph/ijerph-19-13283/article_deploy/ijerph-19-13283-v2.pdf?version=1666681742.

17. McCoy A, Lachman JM, Ward CL, Tapanya S, Poomchaichote T, Kelly J, et al. Feasibility pilot of an adapted parenting program embedded within the Thai public health system. BMC Public Health. 2021;21(1):1009.

18. Redfern A, Cluver LD, Casale M, Steinert JI. Cost and cost-effectiveness of a parenting programme to prevent violence against adolescents in South Africa. BMJ Global Health. 2019;4(3):e001147.

19. Gardner F, McCoy A, Lachman J, Melendez Torres, Tapanya S, Poomchaichote T. Preventing violence against children: Randomised trial of a parenting intervention in the public health system in rural Thailand (forthcoming).

20. Cluver L, Lachman JM, Sherr L, Wessels I, Krug E, Rakotomalala S, et al. Parenting in a time of COVID-19. Lancet. 2020;395(10231):e64.

21. Weisenmuller C, Hilton D. Barriers to access, implementation, and utilization of parenting interventions: Considerations for research and clinical applications. Am Psychol. 2021;76(1):104-15.

22. Lachman JM, Han Q, Juhari R, Ferdinandi I, Raleva M, Jocson R, et al., editors. ParentChat - Preventing Child Maltreatment and Promoting Family Wellbeing Through Online Parent Support Groups in Six Countries. SVRI Forum 2022: the sexual violence research initiative's 7th global conference on violence against woman and violence against children; 2022; Mexico.

23. Department of Economic and Social Affairs. Estimating components of design effects for use in sample design. 2005. In: Household Sample Surveys in Developing and Transition Countries [Internet]. New York: United nations publication; [95-127]. Available from: https://unstats.un.org/unsd/hhsurveys/pdf/household_surveys.pdf.

24. Madans JH, Loeb ME, Altman BM. Measuring disability and monitoring the UN Convention on the Rights of Persons with Disabilities: the work of the Washington Group on Disability Statistics. BMC Public Health. 2011;11(4):S4.

25. Meinck F, Boyes ME, Cluver L, Ward CL, Schmidt P, DeStone S, et al. Adaptation and psychometric properties of the ISPCAN Child Abuse Screening Tool for use in trials (ICAST-Trial) among South African adolescents and their primary caregivers. Child Abuse Negl. 2018;82:45-58.

26. Clerkin SM, Marks DJ, Policaro KL, Halperin JM. Psychometric properties of the Alabama parenting questionnaire-preschool revision. J Clin Child Adolesc Psychol. 2007;36(1):19-28.

27. Frick PJ, Christian RE, Wootton JM. Age Trends in the Association between Parenting Practices and Conduct Problems. Behav Modif. 1999;23(1):106-28.

28. Berry JO, Jones WH. The Parental Stress Scale: Initial Psychometric Evidence. Journal of Social and Personal Relationships. 1995;12(3):463-72.

29. UNICEF. Multiple indicator cluster survey manual, 2005: Monitoring the situation of children and women. New York: UNICEF; 2005.

30. Cianchetti C, Pittau A, Carta V, Campus G, Littarru R, Ledda MG, et al. Child and adolescent behavior inventory (CABI): A new instrument for epidemiological studies and pre-clinical evaluation. Clin Pract Epidemiol Ment Health. 2013;9:51-61.

31. Scott S. Deciding whether interventions for antisocial behaviour work: principles of outcome assessment, and practice in a multicentre trial. Eur Child Adolesc Psychiatry. 2001;10 Suppl 1:I59-70.
32. Henry JD, Crawford JR. The short-form version of the Depression Anxiety Stress Scales (DASS-21): construct validity and normative data in a large non-clinical sample. Br J Clin Psychol. 2005;44(Pt 2):227-39.
33. World Health Organization. WHO multi-country study on women's health and domestic violence against women: Summary report of initial results on prevalence, health outcomes and women's responses. 2005.
34. World Health Organization. Ethical and safety recommendations for intervention research on violence against women. 2016.
35. Janowski R, Cluver LD, Shenderovich Y, Wamoyi J, Wambura M, Stern D, et al. Optimizing Engagement With a Smartphone App to Prevent Violence Against Adolescents: Results From a Cluster Randomized Factorial Trial in Tanzania. J Med Internet Res. 2025;27:e60102.
36. Global Kids Online. Global Kids Online research toolkit: Quantitative tools: Global Kids Online; 2018 [cited 2023 21 October]. Available from: http://globalkidsonline.net/tools/survey/.
37. UNICEF. Multiple Indicator Cluster Surveys (MICS). 2005.
38. Ceballo R, Maurizi LK, Suarez GA, Aretakis MT. Gift and sacrifice: parental involvement in Latino adolescents' education. Cultur Divers Ethnic Minor Psychol. 2014;20(1):116-27.
39. Moncher FJ, Prinz RJ. Treatment fidelity in outcome studies. Clinical Psychology Review. 1991;11(3):247-66.
40. Borrelli B, Sepinwall D, Ernst D, Bellg AJ, Czajkowski S, Breger R, et al. A new tool to assess treatment fidelity and evaluation of treatment fidelity across 10 years of health behavior research. J Consult Clin Psychol. 2005;73(5):852-60.
41. Braun V, Clarke V. Thematic analysis. 2012. p. 57-71.
42. Hollis S, Campbell F. What is meant by intention to treat analysis? Survey of published randomised controlled trials. BMJ. 1999;319(7211):670-4.
43. Morsbach SK, Prinz RJ. Understanding and Improving the Validity of Self-Report of Parenting. Clin Child Fam Psychol Rev. 2006;9(1):1-21.
44. Brewster-Lee D, Lukonge H, Kaaya N, Kagaruki L, Raphael G, Clark A. Faster Reporting, Reducing Errors, and Creating a Community Record: Improving OVC Programming with Mobile Reporting. In: Catholic-Relief-Services E, editor. 2010.
45. Alderson P, Morrow V. Ethics, social research and consulting with children and young people: Ilford : Barnardo's; 2004.
46. Lorenc T, Oliver K. Adverse effects of public health interventions: a conceptual framework. J Epidemiol Community Health. 2014;68(3):288-90.
47. Knerr W, Gardner F, Cluver L. Improving positive parenting skills and reducing harsh and abusive parenting in low- and middle-income countries: a systematic review. Prev Sci. 2013;14(4):352-63.
48. World Health Organization. Researching violence against women : practical guidelines for researchers and activists. Geneva: World Health Organization; 2005.
